# Evaluation of highly conserved *Burkholderia pseudomallei* outer membrane proteins as protective antigens against respiratory melioidosis

**DOI:** 10.1038/s41541-025-01246-2

**Published:** 2025-08-06

**Authors:** Alexander J. Badten, Susana Oaxaca-Torres, Ritwika S. Basu, Matthieu G. Gagnon, Alfredo G. Torres

**Affiliations:** 1 Department of Microbiology and Immunology, University of Texas Medical Branch, Galveston, TX USA; 2 Institute for Translational Sciences, University of Texas Medical Branch, Galveston, TX USA; 3 Department of Biochemistry and Molecular Biology, University of Texas Medical Branch, Galveston, TX USA; 4 Sealy Center for Structural Biology and Molecular Biophysics, University of Texas Medical Branch, Galveston, TX USA; 5 Institute for Human Infections and Immunity, University of Texas Medical Branch, Galveston, TX USA; 6 Department of Pathology, University of Texas Medical Branch, Galveston, TX USA

**Keywords:** Bacteria, Bacteriology, Vaccines

## Abstract

*Burkholderia pseudomallei* (*Bpm*), the etiological agent of melioidosis, lacks approved vaccines. However, several candidates have demonstrated protection in animal models. Interestingly, some of these vaccines can induce cross-protective immunity against the closely related species *B. mallei*. This led us to search the *Bpm* proteome for antigens that are highly conserved in more distantly related pathogenic *Burkholderia* species, which could potentially serve as components of a pan-*Burkholderia* vaccine. We identified three proteins, OmpA1, OmpA2, and Pal, which were coupled to an immunogenic gold nanoparticle (AuNP) platform. Intranasal immunization with these vaccines resulted in the induction of robust Th1/Th2-balanced responses and mucosal immunity, and the AuNP-OmpA1 and AuNP-OmpA2 vaccinated animals were significantly protected from a lethal *Bpm* respiratory challenge. Serum antibodies were highly cross-reactive to *B. mallei* and partially cross-reactive to *B. multivorans* and *B. cenocepacia*, indicating that the antigens contain highly conserved epitopes that can be incorporated in a pan-*Burkholderia* vaccine.

## Introduction

The *Burkholderia pseudomallei* complex comprises mainly two Gram-negative, facultative intracellular bacterial species capable of causing significant human disease: *B. pseudomallei* (*Bpm*) and *B. mallei* (*Bm*)^1,2^. *Bpm*, the etiological agent of the disease melioidosis, is associated with significantly higher global disease incidence, with an estimated 165,000 cases annually, of which 89,000 result in death^3^. While Northern Australia and Southeast Asia have traditionally been recognized as the primary hotspots of melioidosis, modeling studies suggest that *Bpm* is underreported in regions of the Middle East, sub-Saharan Africa, and South and Central America^1,3^. Recently, *Bpm* has been isolated from environmental samples in areas previously thought to be free of the bacteria, including Southern Queensland, Australia^4^ and Mississippi, USA (archive.cdc.gov/www_cdc_gov/han/2022/han00470.html). It has been proposed that climate change is accelerating the bacteria’s spread into new regions, and the increased frequency of severe weather events is expected to correlate with an increase in melioidosis outbreaks^1,4,5^. Furthermore, *Bpm* and *Bm* are still classified by the CDC as Tier 1 select agents due to their potential as biothreat agents (www.selectagents.gov). Despite these concerns, there is currently no approved vaccine available to prevent melioidosis.

In recent years, research into *Bpm* vaccines has made significant strides. Various vaccine formulations have been explored, including live-attenuated strains, outer membrane vesicles derived from *Bpm*, and numerous distinct protein and polysaccharide subunits sourced from *Bpm*^6,7^. Currently, two vaccines are considered leading candidates to prevent melioidosis and are nearing human clinical trials: a subunit vaccine comprising the *Bpm* type 6 secretion system protein Hcp1 paired with the capsular polysaccharide^8^, and *Bpm*-derived outer membrane vesicles that contain a diverse array of antigens^9,10^. Although most vaccination efforts have focused on *Bpm*, some *Bpm* vaccine candidates have demonstrated the ability to elicit cross-protective immunity against both *Bpm* and *Bm*, due to their >99% genetic similarity in conserved genes and shared virulence mechanisms^1,2,9,11–13^. This observation has prompted us to question whether cross-protective immunity could extend beyond the *B. pseudomallei* complex to include more distantly related pathogenic *Burkholderia* species, chiefly the *Burkholderia cepacia* complex (Bcc).

The Bcc consists of over twenty closely related species that are found ubiquitously in the environment worldwide^14^. These opportunistic pathogens are frequently linked to nosocomial outbreaks and are common contaminants in pharmaceutical products due to their inherent resistance to preservatives and nutrient deprivation^14^. Additionally, Bcc members are often isolated from the lungs of cystic fibrosis patients^15^. Such colonization is often refractory to antibiotic treatment, and patients who seem to have cleared the bacteria after treatment may experience a resurgence of infection months or years later^16^. Persistent colonization by Bcc can accelerate decline in lung function^17^ or trigger a rapid onset necrotizing pneumonia, which is typically fatal^18^. In contrast to *Bpm*, only a limited number of groups have explored vaccines targeting Bcc species^19–23^. Therefore, leveraging the interest in vaccines against *Bpm*, we sought to identify antigens that are conserved between *Bpm* and pathogenic Bcc species. Such highly conserved antigens may be capable of eliciting cross-protective immunity to Bcc infection, representing a novel and cost-effective strategy to address this unmet need of CF patients for Bcc preventive measures.

We have previously compiled a comprehensive review of all previously tested *Bpm* complex vaccine antigens, assessing the degree of sequence conservation of each antigen between *Bpm* and the Bcc^7^. While we found little evidence of broad conservation in polysaccharide antigens, a small subset of protein antigens were broadly conserved across the Bcc, with >90% average amino acid similarity^7^. Using an in silico reverse vaccinology screen, we broadened our search for highly conserved *Burkholderia* surface antigens across the entire proteome of *Bpm* strain K96243. Our analysis identified a subset of proteins predicted to be highly antigenic and expressed on the outer membrane, demonstrating a considerable degree of protein sequence conservation among *Bpm*, *Bm*, and two representative members of the Bcc, which are associated with the highest disease incidence and mortality: *B. cenocepacia* and *B. multivorans*^24,25^. Interestingly, three of these antigens were predicted to possess the same OmpA C-like domain, with prior data supporting them as vaccine or therapeutic targets. Consequently, and to confirm our in silico predictions, we opted to conduct head-to-head vaccinations with each of these highly conserved OmpA C-like proteins to evaluate their ability to confer protective immunity against a lethal intranasal (i.n.) challenge of *Bpm* and to elicit cross-reactive antibodies to *Bm*, *B. multivorans*, and *B. cenocepacia*. These studies aim to assist future researchers in determining which, if any, of these antigens merit further investigation for a pan-*Burkholderia* vaccine capable of providing cross-protection against Bcc species. In the current study, we have recombinantly expressed each protein, covalently attached them to a highly immunogenic gold nanoparticle (AuNP) platform designed to elicit robust Th1/Th2-balanced responses and mucosal immunity^12,26–28^, measured vaccine immunogenicity, and assessed the protective efficacy of each vaccine against an i.n. Challenge of *Bpm* strain K96243, and determined the extent of serum antibody cross-reactivity.

## Results

### Reverse vaccinology screen identified outer membrane proteins that are highly conserved among pathogenic *Burkholderia* species

**Fig. 1:**
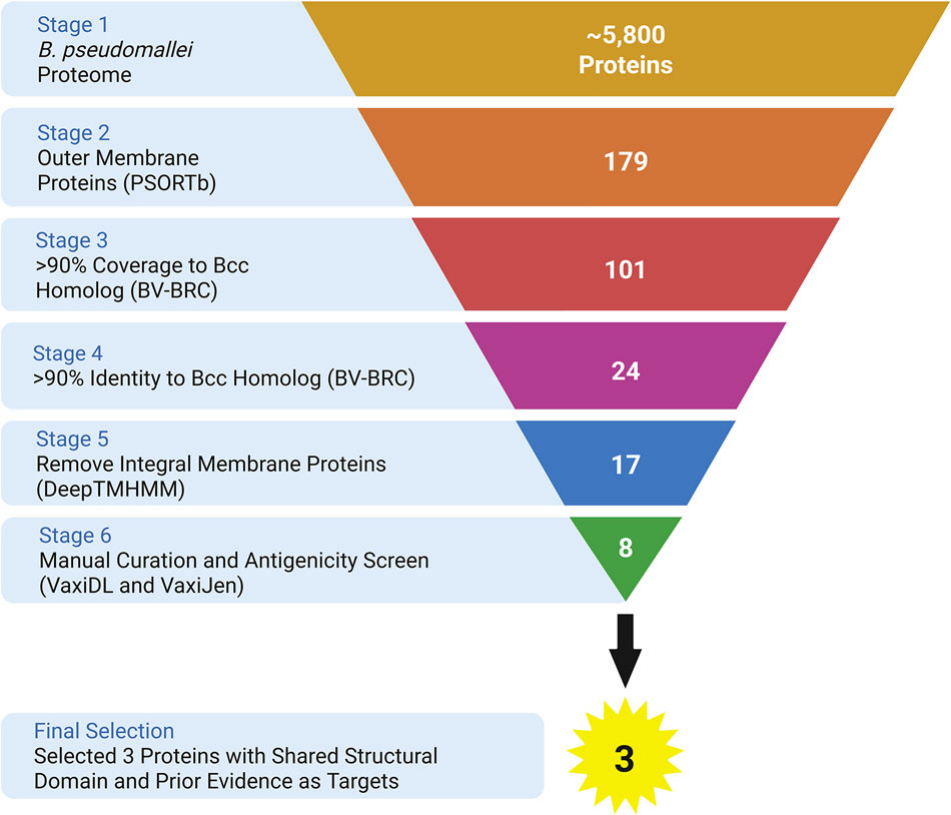
Overview of reverse vaccinology screen to identify highly conserved *Burkholderia* antigens. The *Bpm* K96243 proteome was obtained from RefSeq. Subcellular localization was predicted with PSORTb v3.0.3. The BV-BRC proteome comparison tool (beta) was used to compare sequence conservation and identity of the *Bpm* proteins compared to homologs in *Bm* ATCC 23344, *B. cenocepacia* K56-2, and *B. multivorans* ATCC 17616. Transmembrane topology was predicted with DeepTMHMM v1.0.20. Antigenicity was predicted with Vaxi-DL and VaxiJen. OmpA1, OmpA2, and Pal were selected for further assessment based on their shared structural domain and prior evidence as antigenic vaccination/therapeutic targets. Made with BioRender (https://BioRender.com/p76zt9t).

The complete proteome of the *Bpm* prototype strain K96243 underwent a comprehensive analysis using various bioinformatic screening tools, as detailed in the methods section (Table 1, Fig. 1 and Supplementary Data 1). Initially, proteins that were not predicted^29^ or experimentally confirmed^30^ to be expressed on the outer membrane were excluded from our dataset. This was done to focus our screen on proteins that could potentially be exposed to antibody binding. Subsequently, antigens that were highly conserved between different pathogenic species of the *Bpm* complex (*Bpm* and *Bm*) and the Bcc (*B. cenocepacia* and *B. multivorans*) were selected for further analysis^31^. The remaining proteins were evaluated with the transmembrane topology prediction tool DeepTMHMM v1.0.20^32^, and integral membrane β-barrels were removed due to anticipated technical challenges during recombinant protein expression and subsequent conjugation to the vaccine platform. Finally, we employed Vaxi-DL^33^ and VaxiJen v2.0^34^ to identify proteins predicted to be antigenic. This analysis yielded eight candidates (Table 1).

**Table 1:** *Burkholderia pseudomallei* antigens identified by reverse vaccinology screen

*Bpm* K96243 genomic locus	UniProt-designated protein name	Average sequence coverage (%)	Average sequence identity (%)	VaxiDL prediction score (%)	VaxiJen prediction score	Prior Studies
BPSL0999	OmpA family transmembrane protein	99.2	95.8	99.33	0.8854	^37,38^
BPSL2989	Outer membrane lipoprotein	99.4	94.0	97.7	0.7770	
BPSL2522	Outer membrane protein A	99.3	93.8	82.42	0.5387	^35^
BPSS0294	Multidrug efflux system lipoprotein	98.2	91.1	58.78	0.5234	
BPSS1120	Outer membrane efflux protein	96.9	90.6	90.75	0.5846	
BPSL0816	Outer membrane efflux protein	97.9	90.5	86.83	0.5676	
BPSL1913	Exported protein	98.5	90.4	87.81	0.6578	
BPSL2765	Peptidoglycan-associated lipoprotein	99.4	90.2	68.98	0.5362	^35,36,39^

After a comprehensive review of prior research into these proteins, we identified three candidates with varying degrees of support as vaccine or therapeutic targets: OmpA1 (BPSL0999), OmpA2 (BPSL2522), and Pal (BPSL2765) (Table 1)^35–39^. Notably, all three are predicted to adopt a similar OmpA C-like structure^35^. Among the 12 OmpA C-like domain-containing proteins predicted to be expressed in *Bpm*, OmpA1, OmpA2, and Pal are among the few that have been experimentally detected in the *Bpm* outer membrane, along with BPSL1659^29,30,35^. Interestingly, surface trypsin shaving studies of *B. cenocepacia* have identified homologs of each of these proteins on their surface^40^, where they could be recognized by antibodies. Furthermore, it has been reported that OmpA1, OmpA2, and Pal are some of the most consistently immunodominant antigens during melioidosis infection, with increased antibody levels to all three in human melioidosis patients^35,41,42^. Peptides derived from OmpA1 and OmpA2 also serve as some of the strongest stimulators of peripheral blood mononuclear cells from both seropositive healthy donors and recovered melioidosis patients^43^. This immunodominance largely corroborates the antigenicity predictions made by Vaxi-DL and VaxiJen. Therefore, we selected these proteins as leading candidates for vaccine design.

### In silico and functional characterization of OmpA1, OmpA2, and Pal

**Fig. 2:**
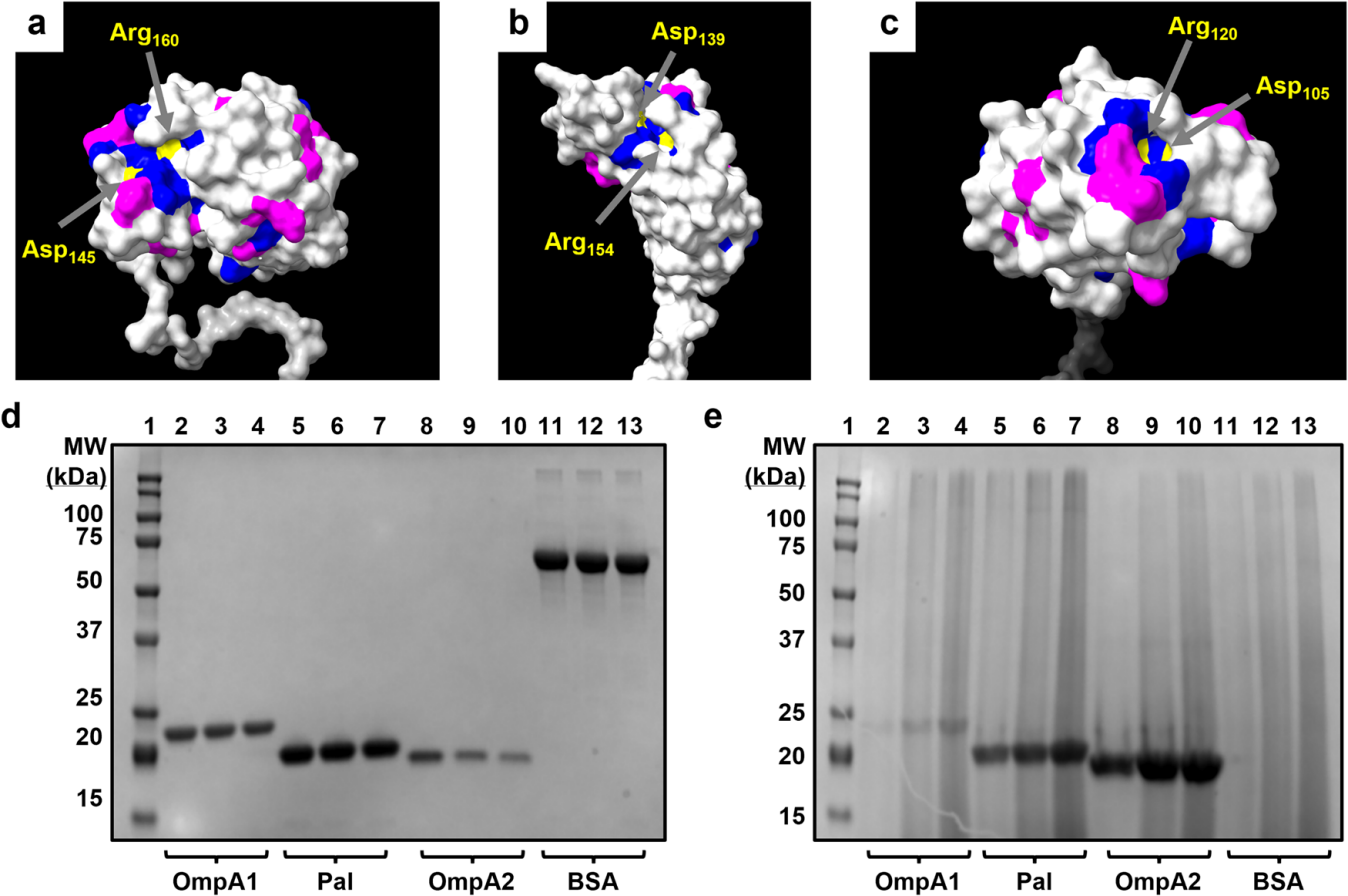
Visualization and functional characterization of conserved peptidoglycan binding domain. ChimeraX v1.6.1 generated structures of the C-terminal regions of OmpA1 (AFDB accession AF-Q63W89-F1) (**a**), OmpA2 (AFDB accession AF-Q63RZ9-F1) (**b**), and Pal (AFDB accession AF-Q63RA7-F1) (**c**)^48,69^. Amino acid residues are colored according to their level of sequence alignment between OmpA1, OmpA2, and Pal as determined by ClustalOmega^47^. Light gray = no major alignment. Purple = the residue at this position has highly similar physiochemical properties across all three proteins. Blue = 100% alignment between all proteins. Yellow = 100% aligned and previously identified as directly involved in peptidoglycan-binding of Gram-negative bacterial OmpA C-like proteins^44^. Coomassie-stained SDS-PAGE gels loaded with supernatant containing unbound protein (**d**) or protein-bound insoluble *E. coli* K12 peptidoglycan (PGN) pellets (**e**) from peptidoglycan-binding assay. 50 µg of either OmpA1 (lanes 2-4), Pal (lanes 5-7), OmpA2 (lanes 8-10), or bovine serum albumin (BSA; lanes 11–13) were incubated with increasing amounts of insoluble PGN (left-to-right: 50, 100, and 200 µg). Lane 1 contains a protein ladder with indicated molecular weight standards. Expected molecular weights: OmpA1 = 20.2 kDa, Pal = 17.5 kDa, OmpA2 = 16.3 kDa, BSA = 66.5 kDa.

As mentioned, each of the three proteins is predicted to adopt an OmpA C-like structure^35^. The OmpA C-like domains, characterized by a β/α/β/α-β(2) motif, resemble the C-terminal domain of *Escherichia coli* OmpA and are known to noncovalently anchor themselves to the peptidoglycan (PGN) layer^44^. Proteins containing such domains typically play a crucial role in maintaining the integrity of the cell envelope structure, as already demonstrated with Pal^36^. However, they may also be involved in virulence-associated functions, including adherence, biofilm formation, and outer membrane vesicle secretion^37,38,45^. Given their predicted shared structural domain, we first aimed to assess the extent of sequence conservation between OmpA1, OmpA2, and Pal. BLASTp comparisons revealed a low degree of primary sequence conservation in their C-terminal regions, with approximately 50-60% total sequence coverage and around 30% sequence identity in each two-way comparison (Fig. S1)^46^. When all three sequences were compared via Clustal Omega^47^, it became apparent that noncontiguous regions in the primary sequence converge to form a surface-exposed patch on the folded protein (Fig. S2 and Fig. 2a–c). Notably, this region contains two amino acids previously identified as essential for PGN-binding in OmpA C-like domains across various Gram-negative bacteria (Fig. 2a–c)^44^. Following the recombinant expression and purification of each protein from the *Bpm* strain K96243 sequences (Fig. S3), we experimentally confirmed their ability to bind to PGN (Fig. 2d, e). Altogether, these findings, along with the AlphaFold^48^ predicted structures, suggest that *Bpm* OmpA1, OmpA2, and Pal are structurally distinct from one another, except for their functional PGN-binding domain.

### Conjugation of OmpA1, OmpA2, and Pal to a gold nanoparticle vaccine platform

**Fig. 3:**
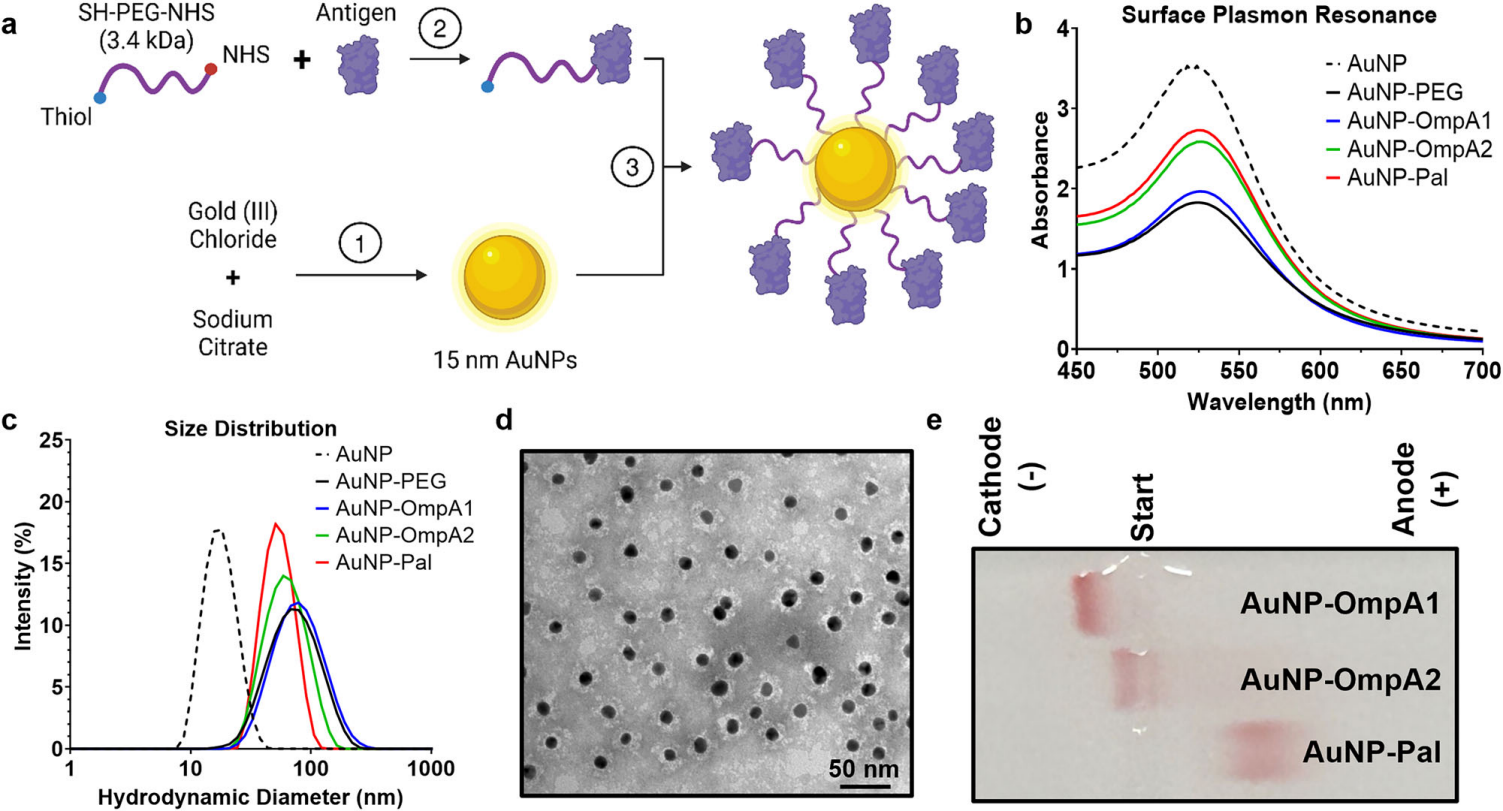
Synthesis and characterization of protein-linked gold nanoparticle vaccines. **a** Schematic illustration of gold nanoparticle vaccine synthesis. Spherical AuNPs were synthesized via the Turkevich method (step 1). The proteins were mixed separately with a heterobifunctional PEG linker, and the NHS end of the linker readily reacts with primary amino groups on the protein (step 2). After purifying the PEGylated protein via size exclusion liquid chromatography, the thiol end of the PEG reacts with surface gold atoms on the AuNPs, resulting in protein-linked AuNPs (step 3). Made with BioRender (https://BioRender.com/l2v5qby). **b** Visible light absorbance measured in 1 nm increments via spectrophotometer. An increase in particle diameter correlates with a slight increase in the wavelength at which maximum absorbance is observed (*λ*_SPR_). **c** Dynamic light scattering measures of Z-average hydrodynamic particle diameter. λ_SPR_ and nanoparticle size distribution values are listed in Table 2. **d** Representative transmission electron microscopy image of AuNP-OmpA2 with uranyl acetate negative staining. **e** AuNP constructs were run on a 0.5% agarose gel at 100 V for ~1 h and then imaged without staining. The TBE running buffer pH was measured to be 8.3, between the predicted isoelectric points of OmpA1/OmpA2 and Pal. ExPASy predicted isoelectric points: OmpA1 = 9.5, OmpA2 = 9.7, Pal = 6.2. Graphs made with GraphPad Prism.

We have previously utilized a gold nanoparticle (AuNP) vaccine platform adjuvanted with CpG ODN 2395 to elicit robust mucosal and Th1/Th2-balanced immune responses to *Bpm* and *Bm* antigens^12,26–28^. The OmpA1, OmpA2, and Pal proteins were each conjugated to this AuNP platform separately using a 3.4 kDa heterobifunctional polyethylene glycol (PEG) linker, featuring a thiol group on one end and an N-hydroxysuccinimide (NHS) group on the other (Fig. 3a). Compared to our earlier conjugation method^12,26–28^, this new approach required fewer steps and resulted in more uniformly coated and stable AuNPs.

First, the optimal reaction ratio of linker-to-protein was experimentally determined for each protein (Fig. S4). After reacting with the linker, the PEGylated proteins were purified using size exclusion liquid chromatography (Fig. S5) before then being reacted with 15 nm spherical AuNPs. The resulting constructs (AuNP-OmpA1, AuNP-OmpA2, and AuNP-Pal) exhibited a marked increase in diameter, primarily attributed to the length of the linker molecule rather than the proteins themselves (Fig. 3b, c and Table 2). Furthermore, we did not observe any visible signs of aggregation during or after the reaction, such as visible solids in the solution or a color shift from red to purple. The absence of aggregation was further confirmed by dynamic light scattering, which did not detect any large particulates in the solution, and visible light spectroscopy, which did not measure a reduction in the A_SPR_/A_450_ ratio (Fig. 3b, c and Table 2). In contrast to unmodified AuNPs (Fig. S6), we observed minimal clustering of conjugated nanoparticles on transmission electron microscopy (TEM) (Fig. 3d), further supporting their effective stabilization by the PEGylated protein. Uranyl acetate negative staining revealed an electron-lucent region surrounding the metallic core of the AuNP, indicative of the presence of PEGylated protein (Fig. 3d). To provide additional evidence that the proteins are stably attached to the AuNPs, we ran the nanoparticles on a 0.5% agarose gel, demonstrating that the gold migrates towards the cathode or anode in accordance with the predicted isoelectric point of the attached protein (Fig. 3e). Mass spectrometry absolute quantitation determined that PEGylated OmpA1 is conjugated to the AuNP at an efficiency of 60.0 ± 13.6% (*N* = 5), corresponding to ~330 ± 95 proteins per nanoparticle. The conjugation efficiency of OmpA2 and Pal was somewhat lower, at 11.2 ± 2.4% (*N* = 5; 58 ± 8 proteins per nanoparticle) for the former and 17.8 ± 10.8% (*N* = 5; 82 ± 43 proteins per nanoparticle) for the latter. Collectively, these results confirm the stability of the nanoparticle constructs and the successful conjugation of the different protein antigens.

**Table 2:** Gold nanoparticle visible light spectroscopy, hydrodynamic size, and ζ potential measures

	Visible Light spectroscopy (Figure 3B)		Zetasizer (Fig. 3C)			
	*λ*_SPR_ (nm)	A_SPR_/A_450_	Hydrodynamic diameter ± SD (nm)	Poly-dispersity Index ± SD	*ζ* Potential ± SD in 0.1X PBS (mV)	*ζ* Potential ± SD in 9 mM Sodium Citrate (mV)
AuNP	518	1.56	16.37 ± 0.31	0.125 ± 0.044	N/A	-41.7 ± 3.85
AuNP-PEG	525	1.56	63.89 ± 1.19	0.250 ± 0.018	-14.6 ± 7.54	-12.4 ± 2.44
AuNP-OmpA1	526	1.66	67.10 ± 1.30	0.251 ± 0.009	-2.38 ± 2.42	-3.82 ± 2.10
AuNP-OmpA2	526	1.67	51.53 ± 1.65	0.230 ± 0.015	-4.09 ± 4.88	-5.18 ± 1.00
AuNP-Pal	524	1.65	48.03 ± 0.33	0.149 ± 0.012	-11.4 ± 2.04	-12.5 ± 1.66

### Characterization of the systemic and mucosal antibody response to vaccination

**Fig. 4:**
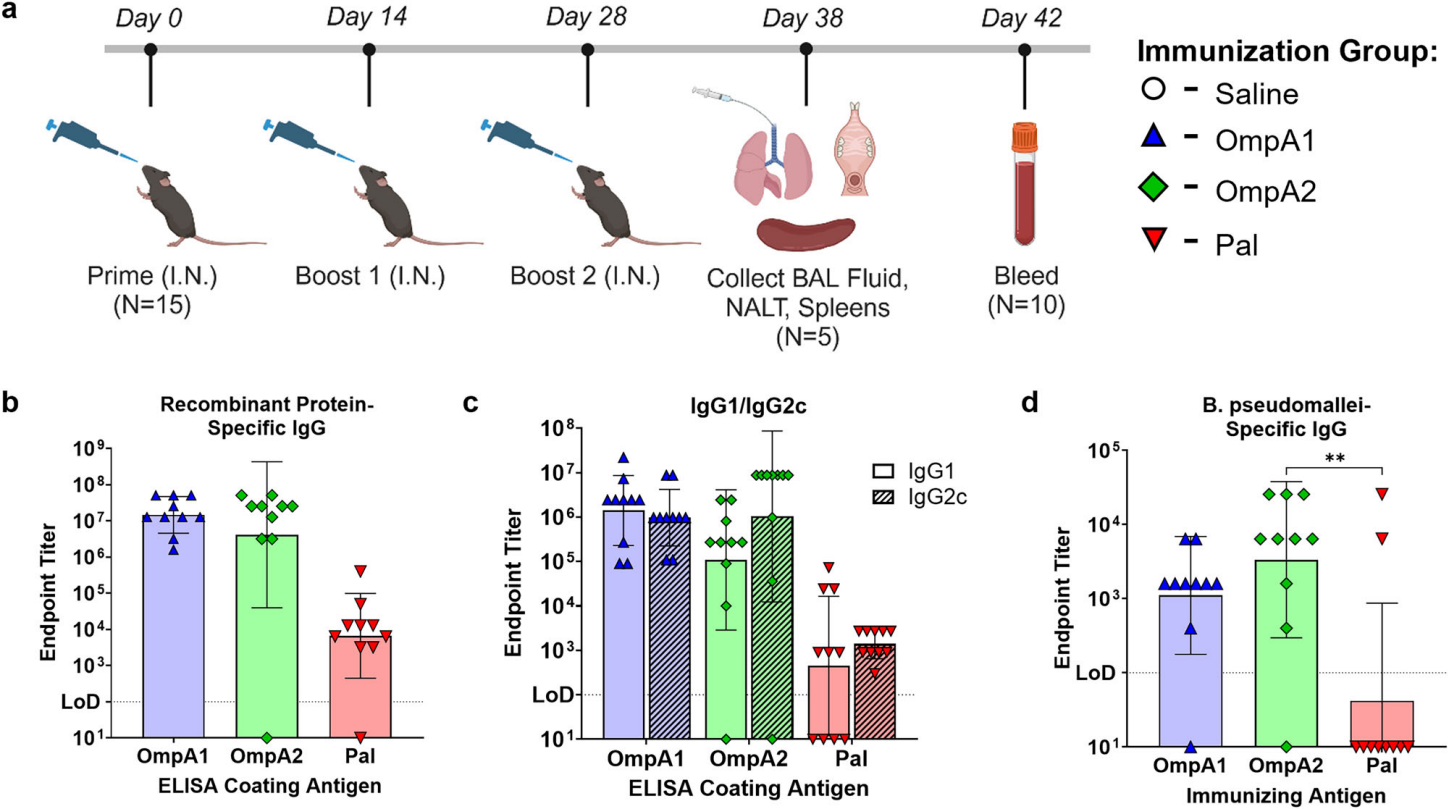
Serum IgG response to vaccination. **a** Timeline of mouse immunizations and tissue collection. Made with BioRender (https://BioRender.com/uxd6kaz). Serial dilutions of serum were probed for total IgG **b** or IgG1/IgG2c **c** via ELISAs coated with the indicated recombinant proteins. **d** The ability of serially diluted serum IgG to react to intact bacteria was measured with ELISA plates coated with *Bpm* strain Bp82. Endpoint titers were calculated as the highest dilution at which measured absorbance was higher than the average absorbance of the equivalently diluted naïve animal serum ± 3 standard deviations. Bacteria-specific IgG endpoint titers were compared via Kruskal–Wallis with Dunn’s multiple comparison test. (**) *p* < 0.01. Graphs made in GraphPad Prism.

To evaluate the humoral immune response to the vaccine candidates, we intranasally (i.n.) immunized C57BL/6 mice three times and collected serum, bronchoalveolar lavage (BAL) fluid, and nasal-associated lymphoid tissue (NALT) at the indicated time points (Fig. 4a). Antigen-specific serum IgG titers in animals vaccinated with AuNP-OmpA1 or AuNP-OmpA2 were approximately three orders of magnitude higher than those in mice immunized with AuNP-Pal (Fig. 4b). However, one of the mice in the AuNP-OmpA2 vaccinated group was entirely unable to mount an antibody response to OmpA2, a finding we have not seen reproduced across four separate immunization experiments. Serum IgG elicited by all three vaccines appeared to be highly IgG1/IgG2c-balanced, indicating a Th1/Th2-balanced response; however, IgG1 levels were below the limit of detection in 4/10 AuNP-Pal vaccinated animals (Fig. 4c). To determine whether these antibodies can react with the natively expressed proteins, we conducted total IgG ELISAs using intact *Bpm* strain Bp82 as the coating material. The OmpA1- and OmpA2-specific antibodies consistently reacted to the bacteria, while only 2/10 AuNP-Pal vaccinated animals exhibited detectable *Bpm*-specific antibodies (Fig. 4d). The inability of Pal-specific antibodies to bind to *Bpm* strain Bp82 could be due to poorer accessibility of Pal compared to OmpA1 and OmpA2, or the result of lower overall antibody titers (Fig. 4b).

**Fig. 5:**
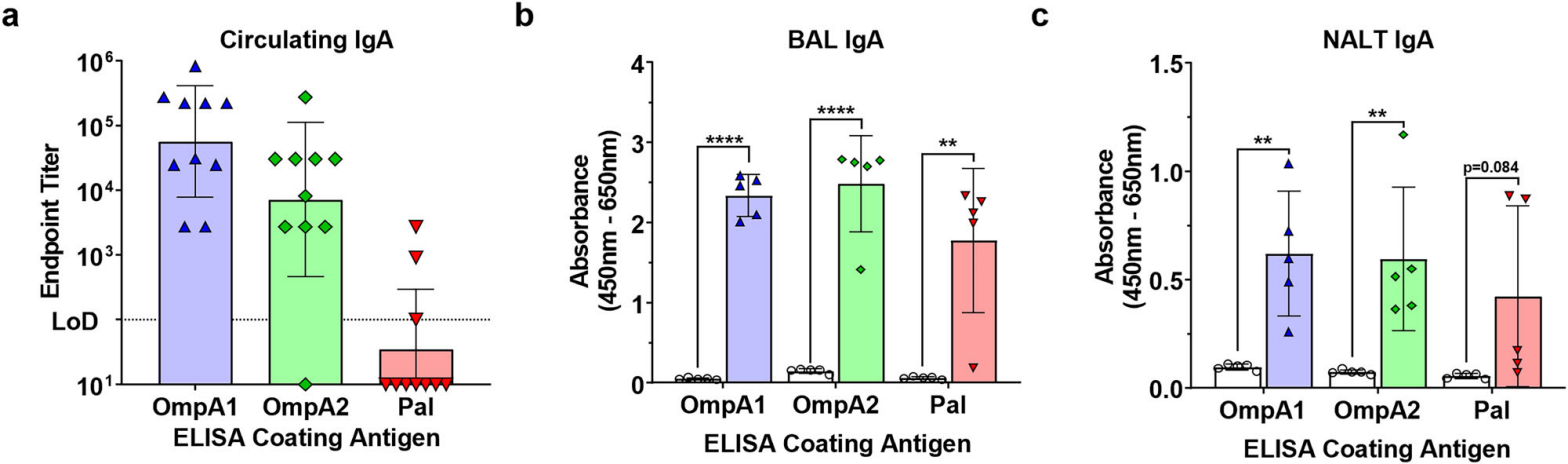
Mucosal IgA response to vaccination. Serial diluted serum (**a**), 1:5 diluted BAL fluid (**b**), and 1:15 diluted supernatants from cultured NALT tissue **c** were probed for antigen-specific IgA. Endpoint titers were calculated as the highest dilution at which measured absorbance was higher than the average absorbance of the equivalently diluted naïve animal serum ± 3 standard deviations. BAL and NALT absorbance values were compared via two-tailed, unpaired Student’s *t*-tests. (**) *p* < 0.01, (****) *p* < 0.0001. Made with GraphPad Prism.

To evaluate mucosal immunity, we first measured serum IgA endpoint titers in the vaccinated animals (Fig. 5a). Once again, animals immunized with AuNP-OmpA1 and AuNP-OmpA2 had consistently higher serum IgA titers than animals immunized with AuNP-Pal, of which only 3/10 animals showed detectable circulating IgA levels (Fig. 5a). Interestingly, when we probed BAL fluid from a different cohort of animals for secreted IgA, we observed that all vaccinated animals had comparably high IgA levels, except for one weak responder in the AuNP-Pal group (Fig. 5b). Additionally, we measured levels of secreted IgA in supernatants obtained from NALTs that had been cultured in complete RPMI for 24 h (Fig. 5c). Although NALT-secreted IgA varied somewhat between animals, all immunized animals had detectable NALT-produced IgA (Fig. 5c). Furthermore, compared to the other two groups, AuNP-Pal vaccinated animals exhibited a more bimodal distribution in NALT IgA secretion, characterized by two strong responders and three of the weakest overall responders (Fig. 5c). Altogether, these findings indicate that the AuNP-OmpA1 and AuNP-OmpA2 vaccines consistently elicited robust levels of systemic IgG, circulating IgA, and secreted IgA. In contrast, animals immunized with AuNP-Pal had lower IgG, minimal circulating IgA, and inconsistent secreted IgA. Given these tissue-specific differences, we hypothesize that the pharmacokinetics of AuNP-Pal may differ from those of AuNP-OmpA1 and AuNP-OmpA2. Because these proteins are largely comparable in terms of size and structure yet exhibit considerable differences in their isoelectric points (Fig. 3e and Table 2), we further theorize that the more anionic nature of AuNP-Pal may be responsible for these differences.

### Evaluation of the induction of systemic T cell responses by AuNP vaccination

**Fig. 6:**
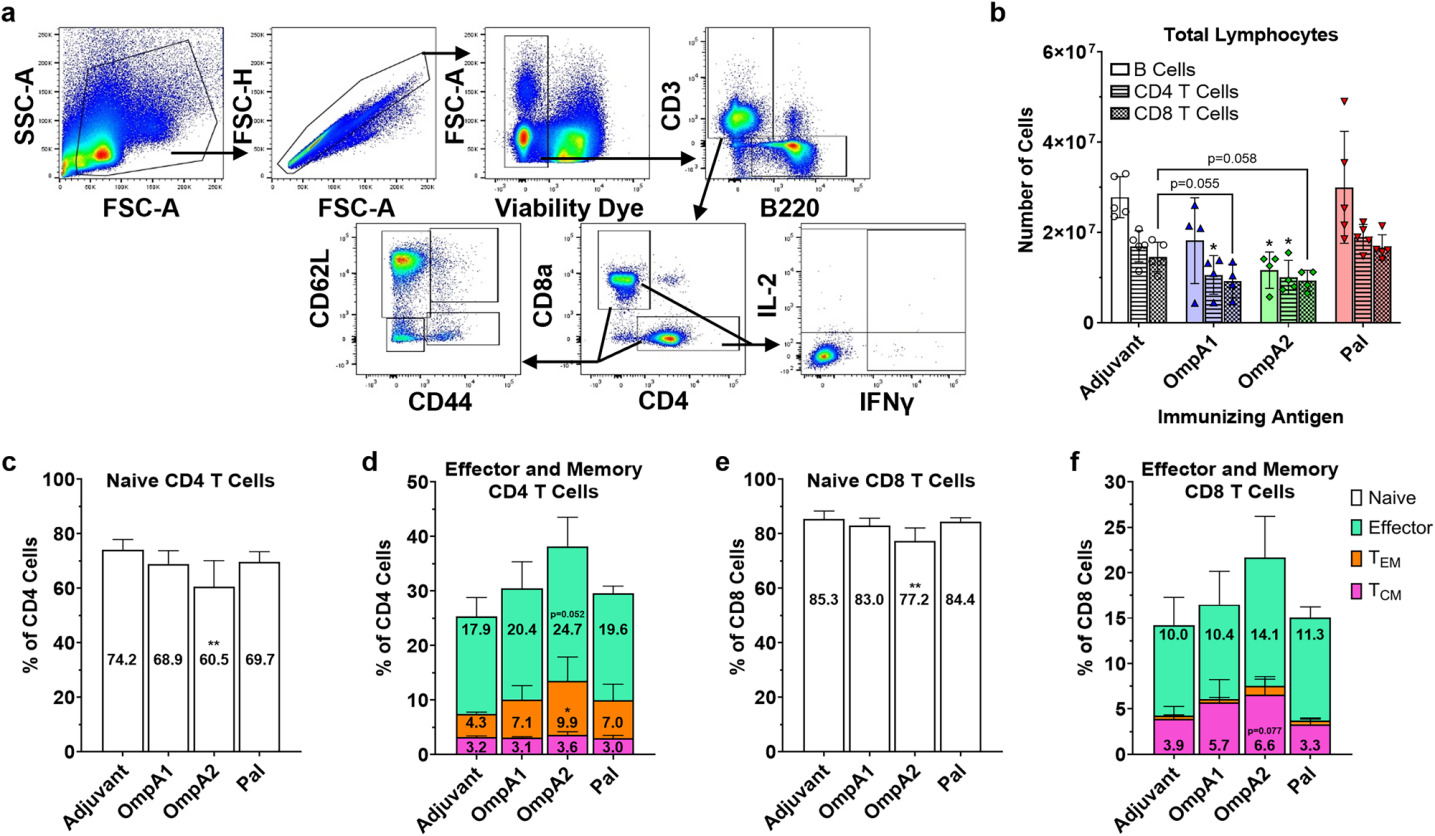
Splenic lymphocyte response post vaccination. **a** Flow cytometry gating strategy. **b** Total lymphocyte counts recovered from vaccinated animals. B cells = B220 + CD3-, CD4 T cells = CD3 + CD4 + CD8a-, and CD8 T cells = CD3 + CD4-CD8a + . CD44 and CD62L expression patterns of CD4 T cells **c**, **d** and CD8 T cells (**e**, **f**), measured via flow cytometry. Naïve cells = CD44-CD62L + , effector cells = CD44 − CD62L−, effector memory cells (*T*_EM_) = CD44 + CD62L−, and central memory cells (T_CM_) = CD44 + CD62L+. Total lymphocyte counts and proportions of CD44/CD62L-expressing T cells were compared via one-way ANOVAs with Dunnett’s multiple comparison correction. (*) *p* < 0.05, (**) *p* < 0.01. Made with GraphPad Prism.

We measured broad changes in splenic lymphocytes collected 10 days post-vaccination via flow cytometry (Fig. 6a). Counterintuitively, spleens collected from AuNP-OmpA1 and AuNP-OmpA2 vaccinated animals had markedly fewer lymphocytes than those collected from mice immunized with an adjuvant control or AuNP-Pal (Fig. 6b). Given the time at which the spleens were collected, we speculate that the spleens had already gone through the expansion phase in response to the vaccine, and the reduced lymphocytes counts could be attributed to the spleens entering the contraction phase in which most of the expanded lymphocytes undergo apoptosis^49^. Alternatively, or in concert, the reduced splenic lymphocytes could also be attributed to egress of the cells to other tissues^50^. We also assessed CD4 and CD8 T cell phenotypes by staining for CD44 and CD62L (Fig. 6c–f). The AuNP-OmpA2 vaccinated group consistently showed the most marked differences in T cell phenotypes, exhibiting statistically significant reductions in naïve CD4 and CD8 cells, and correspondingly increased proportions of effector cells, effector memory cells (T_EM_), and central memory cells (T_CM_) (Fig. 6c–f). The spleens from both the AuNP-OmpA1 and AuNP-Pal groups trended in the same direction but fell short of reaching statistical significance (Fig. 6c–f). Notably, while the AuNP-OmpA1 and AuNP-Pal groups exhibited highly similar T cell phenotype distributions, the AuNP-Pal vaccinated animals had lower CD8 T_CM_ cells, potentially indicating that a less durable CD8 T cell response had been elicited (Fig. 6f).

**Fig. 7:**
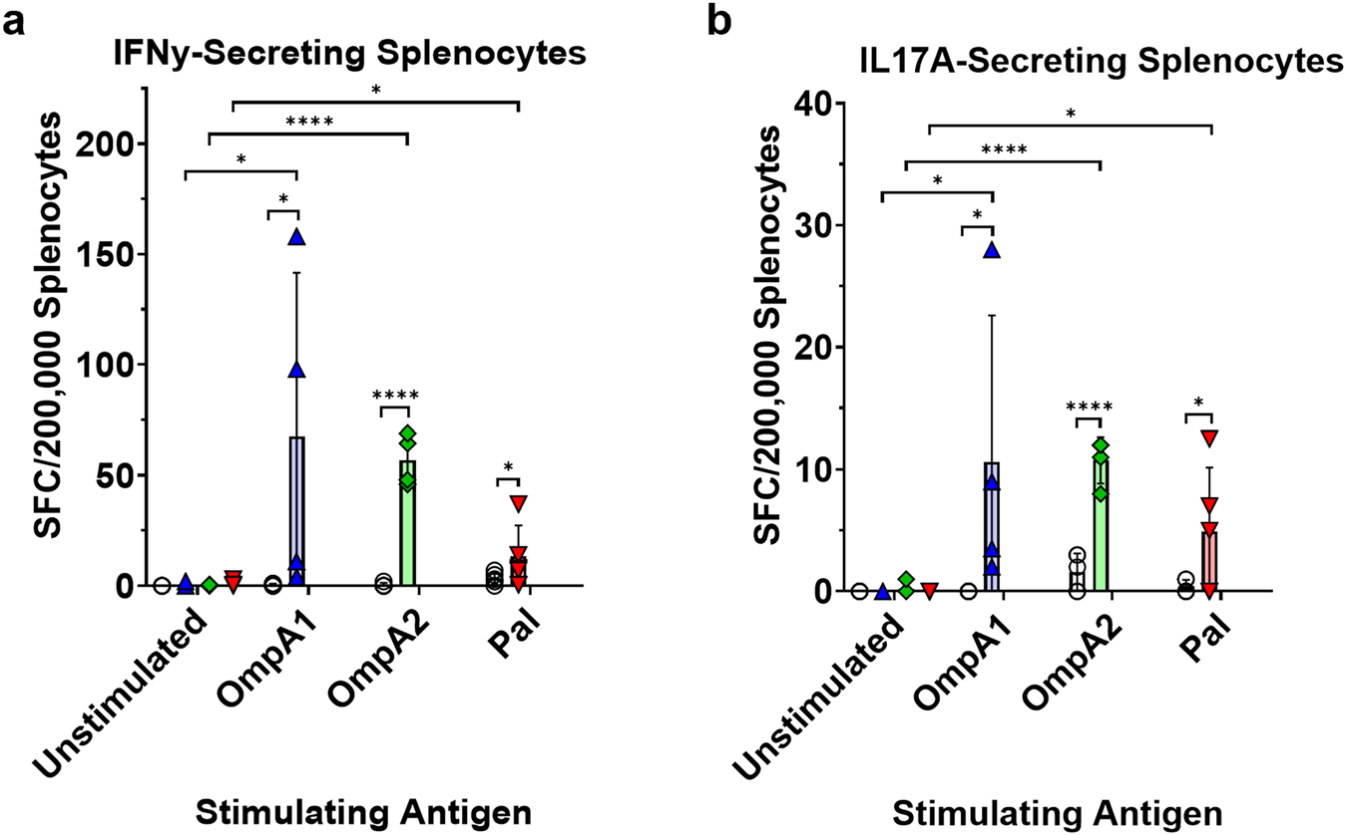
Splenic T cell recall response. Measure of IFNγ-producing splenocytes after 24 h of stimulation **a** or IL-17A-producing splenocytes after 48 h of stimulation **b** with the indicated antigen, as measured via ELISpot. The proportions of IFNγ- or IL-17A-secreting splenocytes were compared via matched-pairs two-way ANOVAs with Fisher’s LSD tests. (*) *p* < 0.05, (****) *p* < 0.0001. SFC = spot-forming cells. Made with GraphPad Prism.

Next, we conducted ex vivo stimulations of splenocytes using the recombinant proteins to evaluate the magnitude and phenotype of the T cell response. Splenocytes from the immunized groups consistently produced IFNγ in response to the antigen stimulation (Fig. 7a). The AuNP-OmpA1 and AuNP-OmpA2 groups had significant increases in the proportion of IFNγ-producing CD4 T cells, whereas only the latter had a significant increase in IFNγ-producing CD8 T cells (Fig. S7). Again, the AuNP-Pal group appeared to elicit a weaker, albeit detectable, response (Fig. 7a and Fig. S7). Similarly, recalled splenocytes from all groups produced IL-17A (Fig. 7b), which is often associated with the establishment of mucosal immunity^51^. We were unable to detect IL-4-producing cells either by flow cytometry or ELISpot. Given the IgG1/IgG2c-balanced response previously observed (Fig. 4c), we speculate that either the time point at which spleens were collected was not optimal for detecting IL-4-producing Th2 cells, or that Th2 cells elicited by our vaccines primarily localize to other secondary lymphoid tissues, such as the draining lymph nodes around the lungs or within the NALT. Altogether, this data supports the idea that AuNP-OmpA2 and AuNP-OmpA1 elicited strong systemic T cell responses, largely correlating with the antibody data. Furthermore, we were able to confirm the Th1- and Th17-stimulating properties of our vaccine platform.

### OmpA2 confers significant protection against a lethal intranasal *Bpm* challenge in C57BL/6 Mice

**Fig. 8:**
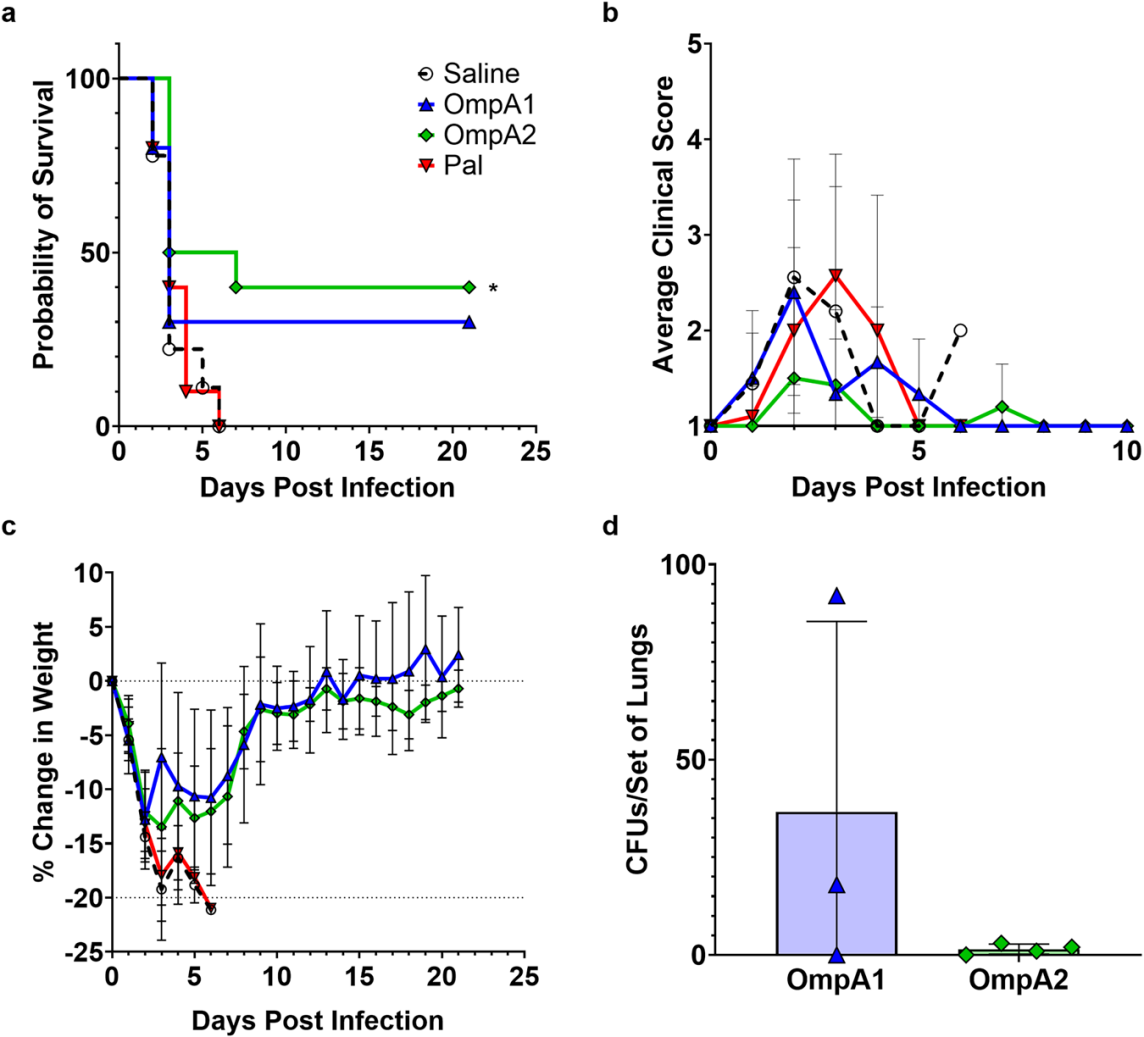
Vaccine efficacy against a lethal intranasal challenge of *Bpm* strain K96243. After receiving the full immunization regimen, mice were challenged i.n. with 2.2 × LD50 of Bpm K96243. Lungs, livers, and spleens from animals that survived to 21 dpi were homogenized, serially diluted, and plated on Ashdown plates to assess persisting organ colonization. **a** Kaplan–Meier survival curves. **b** Average group clinical scores up to 10 dpi. All clinical scores recorded past 10 dpi were 1. Clinical scores were reported as follows: 1 = active and healthy appearance; 2 = mild lethargy; 3 = ruffled fur, hunched posture, and mild lethargy; 4 = ruffled fur, hunched posture, limited mobility; 5 = moribund. **c** Percent change in weight from 0 dpi. **d** Colony-forming units (CFU) cultured from lungs at 21 dpi. Kaplan–Meier curves of the vaccinated groups were compared to the saline control via log-rank tests, and adjusted *p* values were calculated using the Bonferroni multiple comparison correction. (*) *p* < 0.05. Made with GraphPad Prism.

The C57BL/6 mice were immunized as described (Fig. 4a) and subsequently challenged 3 weeks after the last immunization with 2.2 × LD_50_ of *Bpm* strain K96243. Over the course of the first 7 days post-infection (dpi), all naïve animals and AuNP-Pal-vaccinated animals succumbed (Fig. 8a). In contrast, 3/10 and 4/10 animals survived acute infection in the AuNP-OmpA1 and AuNP-OmpA2 groups, respectively. No deaths were observed in the chronic phase of infection (>7 dpi) (Fig. 8a). During the first week post-infection, the AuNP-OmpA2-vaccinated group appeared to have consistently lower clinical scores (Fig. 8b). 7/10 animals in the AuNP-OmpA2 group never received clinical scores higher than 2, compared to the other groups, which each had only 3 animals with such consistently low clinical scores. However, despite these apparent differences in clinical scores, all groups experienced consistent weight loss in the first 7 dpi (Fig. 8c). When lung, liver, and spleen suspensions were cultured for bacteria at 21 dpi, organs from the AuNP-OmpA2 group were virtually sterile, with only 0–3 CFUs found in the lungs of surviving animals (Fig. 8d). By comparison, 2/3 of the remaining AuNP-OmpA1 vaccinated animals had higher remaining lung colonization at 61 and 100 CFUs per set of lungs, suggesting that OmpA1 expression is lower during the chronic phase of disease (Fig. 8d). We also found that spleens recovered from the AuNP-OmpA2 vaccinated mice were slightly enlarged compared to those from the AuNP-OmpA1 vaccinated mice (*p* = 0.0663), potentially indicating a stronger or more prolonged immune response to the challenge (Fig. S8). Overall, these data provide evidence that OmpA2, and to a lesser extent OmpA1, are protective as individual antigens against *Bpm*.

We also explored whether protection could be improved by administering a higher dose of vaccine. We immunized C57BL/6 mice again according to Fig. 4a, this time using four times the concentration of nanoparticles per dose and a constant amount of CpG ODN 2395 adjuvant. Unfortunately, reactogenicity was observed after the second or third immunization in half of the animals receiving the higher dose of AuNP-Pal or AuNP-OmpA2. Dosing studies with AuNP-OmpA2 suggest that this reactogenicity may be related to an excessive T cell response, which correlated with vaccine dose (Fig. S9), and not to the antibodies, which remained unchanged (Fig. S10). However, when the surviving animals were challenged with a higher dose of 5.0 × LD_50_ of *Bpm* strain K96243, 3/4 animals in the AuNP-OmpA2 group survived to 21 dpi, a statistically significant improvement compared to the saline and adjuvant control groups, in which none survived (Fig. S11a). None of the AuNP-OmpA1 vaccinated animals and only 1/4 AuNP-Pal animals survived to the study endpoint, though the AuNP-OmpA1 group tended to succumb slightly later than the control groups (Fig. S11a). All the AuNP-OmpA2 vaccinated animals appeared active and healthy for the duration of the challenge study, with none reporting clinical scores higher than 1 (Fig. S11b). In contrast, 8/8 naïve animals, 7/8 adjuvant control animals, 5/8 AuNP-OmpA1 animals, and 3/4 AuNP-Pal animals exhibited overt signs of infection (Fig. S11b). Despite improved clinical scores, all groups once again exhibited consistent weight loss in response to the challenge, though the AuNP-OmpA2 group had a slight but statistically significant (*p* = 0.012) improvement at 1 dpi compared to the saline control group (Fig. S11c). At the study endpoint of 21 dpi, we found that all remaining animals had 15 to 36 CFUs remaining in the spleens and lungs, and between 600 and 4200 CFUs in the livers (Fig. S11d–f). We attribute the higher remaining organ colonization in this study to the higher initial challenge dose. Therefore, vaccination with the OmpA2 protein still elicited significant protection from a higher dose of *Bpm* strain K96243, confirming its protective properties and providing evidence that a higher dose of vaccine may elicit more robust protection if reactogenicity can be eliminated.

### OmpA1, OmpA2, and Pal antibodies exhibit varying degrees of cross-reactivity to *B. mallei*, *B. multivorans*, and *B. cenocepacia*

**Fig. 9:**
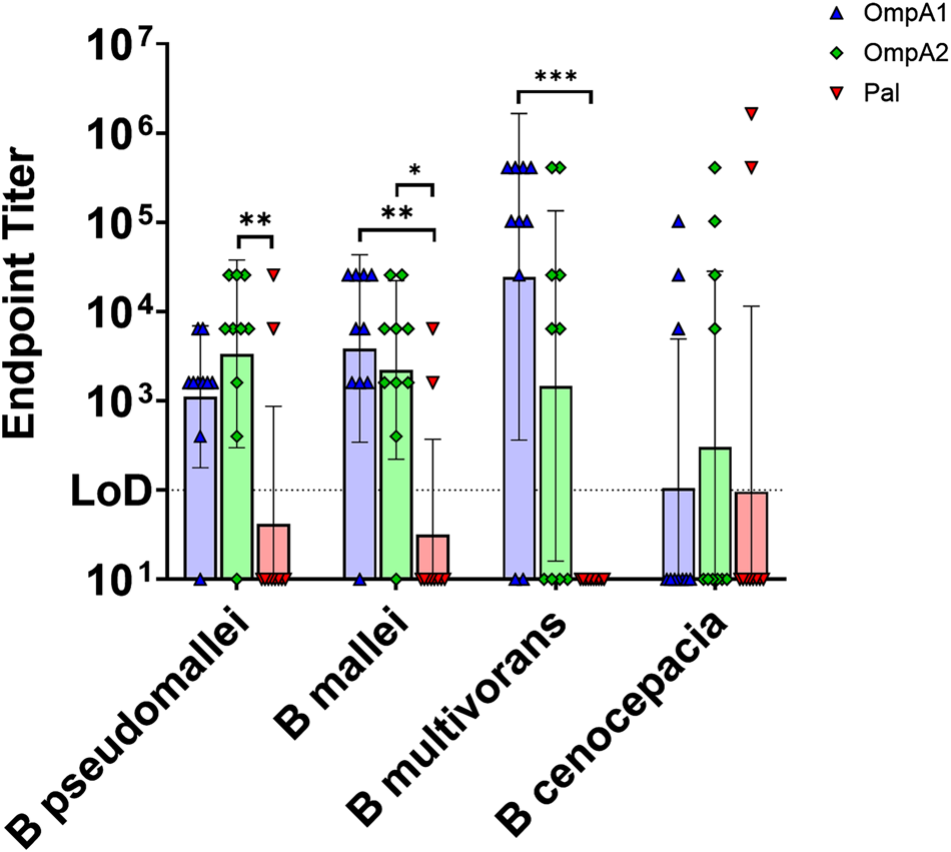
Measuring IgG cross-reactivity to heterologous *Burkholderia* species. Serial dilutions of immunization serum were probed for IgG capable of binding to *Bm* strain CLH001, *B. multivorans* strain ATCC 17616, or *B. cenocepacia* strain K56-2 via ELISAs coated in the indicated bacteria. The *Bpm* strain Bp82 ELISA results from Fig. 4d are included for side-by-side comparison. Endpoint titers were calculated as the highest dilution at which measured absorbance was higher than the average absorbance of the equivalently diluted naïve animal serum ± 3 standard deviations. Endpoint titers were compared via Kruskal–Wallis with Dunn’s multiple comparison test. (*) *p* < 0.05, (**) *p* < 0.01, (***) *p* < 0.001. Made in GraphPad Prism.

We next probed the mouse vaccination serum against intact *Bm* strain CLH001, *B. multivorans* strain ATCC 17616, and *B. cenocepacia* strain K56-2 (Fig. 9). Serum reactivity to *Bm* strain CLH001 was largely comparable to reactivity against *Bpm* strain Bp82 for all three vaccination groups, indicating that protein expression, location, and sequence conservation were largely conserved between *Bpm* and *Bm* (Fig. 9). Cross-reactivity to *B. multivorans*, a more distantly related species within the Bcc, was readily detected with 8/10 and 6/10 animals in the AuNP-OmpA1 and AuNP-OmpA2 groups, respectively, exhibiting high, detectable titers (Fig. 9). *B. cenocepacia* cross-reactivity was much less consistent, with just 3/10 responders in the AuNP-OmpA1 group and 4/10 in the AuNP-OmpA2 group. In contrast, the two *Bpm*- and *Bm*-reactive animals in the AuNP-Pal vaccination group exhibited particularly strong reactivity to *B. cenocepacia* but did not react at all to *B. multivorans*, potentially indicating that the immunodominant epitope(s) being recognized by these animals are not accesible or conserved in *B. multivorans* (Fig. 9). Notably, endpoint titers did not gradually decrease when using the more distantly related species of bacteria. Instead, we observed an “all-or-nothing” response whereby animals either exhibited high cross-reactive endpoint titers or no detectable cross-reactivity. Overall, this data confirms that highly cross-reactive antibodies can be elicited to these proteins, albeit in a potentially epitope-dependent manner.

## Discussion

Using a reverse vaccinology approach that prioritizes *Burkholderia* interspecies sequence conservation, we identified several highly conserved, outer membrane-expressed proteins that were predicted to be antigenic (Fig. 1 and Table 1). A noteworthy protein in this list, which has not yet been explored, is BPSL2989, which exhibits homology to *E. coli* SlyB. Interestingly, SlyB homologs have been frequently identified as potential vaccine antigens in other pathogenic Gram-negative species^52–54^. Additionally, it should also be noted that we removed seven highly conserved proteins from our analysis due to their predicted integral membrane topology—a purely technical reason. However, such integral membrane proteins have frequently been explored as *Bpm* and *Bm* vaccine antigens in past studies^7,12,21,27,28,55^. One such protein that was removed from our analysis despite having some of the highest overall sequence conservation was BPSL2151, the *Bpm* homolog of BamA. BPSL2151 has previously been characterized as a protective antigen in intraperitoneal models of *Bpm* infection, highlighting that such integral membrane proteins may serve as valuable targets for vaccination^55^. If such proteins are added back to our list of highly conserved vaccine antigens (Table 1), then we have identified as many as 15 targets for future study. These 15 proteins are broadly conserved between *Bpm*, *Bm*, *B. cenocepacia*, and *B. multivorans*, making them optimal targets for a theoretical pan-*Burkholderia* vaccine.

Of the three OmpA C-like proteins we pursued in this work, only Pal has been functionally characterized as playing a role in maintaining cell envelope integrity^36^. By comparison, little is known about the functions of *Bpm* OmpA1 and OmpA2. In *B. cenocepacia* and *B. multivorans*, it appears that OmpA1 may be involved in biofilm formation and adherence to lung epithelial cells^37,38^, though similar work has yet to be conducted in *Bpm* or *Bm*. By comparison, we found little information about the function of OmpA2, though it is unique in that it is essential to both *B. cenocepacia* and *Bpm* survival^56,57^. While functionally characterizing these proteins was outside the scope of this work, we were able to confirm their peptidoglycan (PGN)-binding ability (Fig. 2d, e). Interestingly, OmpA1 appeared to have a significantly lower ability to bind PGN compared to Pal and OmpA2. The closest homolog of OmpA1 in *Pseudomonas aeruginosa*, PA0833, was found to have an enlarged PGN binding pocket, which affects its PGN-binding properties^58^, potentially explaining our finding. Regardless, a more thorough investigation of the functions of OmpA1 and OmpA2 is needed.

To assess the protective role of these antigens in an established murine model of melioidosis, we first successfully conjugated the recombinant proteins to an AuNP platform that we have previously utilized to induce robust mucosal and Th1/Th2-balanced immune responses^12,26–28^. However, we made improvements to our conjugation scheme to simplify the process, increase efficiency, and better stabilize the nanoparticles (Fig. 3). The most noteworthy modification to our synthesis scheme was changing the backbone of our linker molecule from the short, relatively hydrophobic carbon chain in 16-mercaptohexadeconic acid (16-MHDA) to a much longer and more hydrophilic backbone consisting of PEG. We empirically found that this linker improved stability and shelf-life of our vaccines, likely due to the more hydrophilic nature of the linker and its ability to sterically hinder the metallic AuNP cores from directly interacting with each other, thereby preventing aggregation. Additionally, PEG is much more widely adopted as a linker in the field of biotechnology than 16-MHDA^59^, and it has been determined that PEGylated nanoparticles can effectively penetrate mucus in the lungs for better drug delivery^60^. The second noteworthy change to our synthesis protocol is that the new linker contains an NHS functional group, which can immediately react with primary amino groups on proteins at high efficiency. By comparison, the 16-MHDA linker in our past studies had a carboxylic acid functional group, which had to be activated as an additional step with either 1-ethyl-3-(3-dimethylaminopropyl)carbodiimide (EDC) followed by NHS^26^, or just 4-(4,6-dimethoxy-1,3,5-triazin-2-yl)-4-methyl-morpholinium chloride (DMTMM)^12,27,28^. By removing this activation step, we were able to simplify the protocol, increase conjugation efficiency, and reduce the likelihood of off-target reactions. These changes should increase the accessibility of this technology for other researchers.

In the context of intracellular bacterial pathogens such as *Bpm*, it is generally accepted that a balanced Th1/Th2 response is critical for controlling the intracellular and extracellular phases of infection, respectively^61^. In characterizing the response to our vaccines, we found that a robust Th1 response was elicited by the vaccines (Figs. 4c, 7a and S7a, S9a, c). Studies in human melioidosis patients have clearly demonstrated that such Th1 responses correlate with survival^62^. Th2 responses were also likely generated, based on the high IgG1 titers detected (Fig. 4c); however, we were unable to directly detect Th2 cells in the spleen 10 days after receiving the full vaccination regimen, possibly due to the collection time or because Th2 cells were restricted to other secondary lymphoid tissues nearer the site of immunization. When we further characterized the T cell response in spleens of mice that had been vaccinated 10 days prior, we found a reduced proportion of naïve CD4 and CD8 T cells, and corresponding increases in effector T cells, T_EM_ cells, and T_CM_ cells (Fig. 6c–f). The induction of T_EM_ and T_CM_ cells is indicative of a durable T cell response necessary for long-term immunity. We also observed an induction of Th17 cells (Figs. 7b and S9b, d) and increased production of antigen-specific IgA (Fig. 5) after vaccination. Such responses are important components of mucosal immunity, which is considered advantageous for controlling *Bpm* since they are frequently acquired by the respiratory route^1,12^. Therefore, our AuNP vaccine platform, paired with CpG ODN 2395, elicited strong humoral and cellular immune responses associated with protection in melioidosis.

In our vaccine immunogenicity studies, we found that the AuNP-OmpA1 and AuNP-OmpA2 vaccines consistently elicited robust humoral and T cell responses, whereas AuNP-Pal responses were of a lower magnitude and were unable to confer protection. This was an unexpected finding given that Pal has received the most prior attention as a potentially protective antigen against both *Bpm*^35,39^ and *Bm*^36^. We postulated two potential explanations for the weaker immune response elicited by AuNP-Pal. First, in all previous studies of *Bpm* and *Bm* Pal as a vaccine antigen, the authors consistently used the BALB/c strain of mice^35,36^, whereas we primarily used C57BL/6 for our studies. Interestingly, when we attempted to immunize BALB/c mice with the same vaccine regimen, we found that all animals had endpoint IgG titers between 10^5^ and 10^6^ (unpublished data), which was higher on average than the C57BL/6 Pal titers (Fig. 4b). Given their different MHC haplotypes and genetic backgrounds, these strains may be expected to have different naïve T cell and/or B cell receptor repertoires that allow BALB/c to respond to the antigen more readily. Second, while all three proteins have similar structures and sizes, OmpA1 and OmpA2 noticeably differ from Pal in their isoelectric points (pI). Pal’s slightly acidic pI (predicted 6.2), in contrast to the highly basic pI values of OmpA1 and OmpA2 (predicted 9.5 and 9.7, respectively), confers a more anionic surface charge to the AuNPs, as we experimentally confirmed (Table 2 and Fig. 3e). It has been demonstrated that cationic nanoparticles are more disruptive to cellular membranes, which may serve to increase the adjuvanticity of the vaccine by activating patterns recognition receptors such as NLRP3 that mediate inflammation in response to such damage^63^. Furthermore, cationic nanoparticles are taken up by nonphagocytic cells more efficiently than anionic particles, potentially resulting in stronger CD8 T cell responses^64^, which we observed (Fig. 6e, f). Additionally, surface charge affects the ability of nanoparticles to penetrate the lung mucus^65^. Therefore, the reduced immunogenicity of AuNP-Pal, and consequently its lower protective efficacy, may stem from issues associated with the animal model or potential surface charge-related effects on pharmacokinetics and pharmacodynamics.

Very promisingly, AuNP-OmpA1 and AuNP-OmpA2 elicited robust humoral and cellular immune responses that were capable of inducing protection to lethal challenges of *Bpm*. In addition to their superior immunogenicity, we also found that IgG elicited to OmpA1 and OmpA2 could react to a lab-attenuated strain of *Bpm*, providing direct evidence that vaccine-induced antibodies can react to the native protein on the surface of the bacteria. Given that the OmpA2 vaccine elicited more consistent protection than the OmpA1 vaccine despite comparable humoral and T cell responses, it is possible that OmpA2 is expressed at a higher level or more constitutively during infection. Recently, Heacock-Kang et al. assessed *Bpm* transcriptomics across different stages of intracellular infection compared to bacteria grown in vitro^66^. The authors did not report BPSL2522 (OmpA2) as differentially expressed, potentially indicating relatively uniform expression. By comparison, BPSL0999 (OmpA1) was significantly upregulated during the cytoplasmic phase of infection, and BPSL2765 (Pal) was upregulated during membrane protrusion^66^, indicating that expression of these proteins may be more temporally regulated. One might predict that an antigen that is uniformly expressed during all stages of infection would make a better target for vaccination; however, more work is needed to confirm if translated protein levels match this transcriptomic data.

While sterilizing immunity and full protection were not achieved with any of the antigens on their own, AuNP-OmpA2 conferred a statistically significant reduction in mortality and caused a clear reduction in overt signs of infection, making it a highly promising target for future vaccination studies. Our own experience testing multiple antigens has taught us that very few subunit vaccines are able to show statistically significant protection using individual protein antigens in respiratory models of melioidosis, and instead, it has primarily been the surface polysaccharide antigens that mediate near-complete protection in such models^7,8,12,26–28^. Therefore, a logical next step may be to pair OmpA2 with these protective polysaccharide antigens, namely the capsular polysaccharide^8^ or the lipopolysaccharide O-antigen^12,26–28^, to determine whether they display a synergistic effect and result in a fully protective melioidosis vaccine. Furthermore, it has been shown that a leading melioidosis vaccine candidate consisting of *Bpm*-derived outer membrane vesicles contains high levels of OmpA2^10^. Therefore, it may be worth exploring to what extent OmpA2 is responsible for the protection observed in this vaccine.

Finally, since the original goal of this research was to identify antigens that are conserved across pathogenic *Burkholderia* species, we also demonstrated that antibodies elicited to the *Bpm* proteins exhibit cross-reactivity to more distantly related *Burkholderia* species. Serum antibodies from the vaccinated animals responded to *Bm* at essentially the same level as to *Bpm* (Fig. 9), an expected finding given the high degree of genomic conservation between these species^1,2,13^. Future work may further explore whether *Bpm*-derived OmpA1 and OmpA2 can protect against a heterologous *Bm* challenge. We also found that a subset of the AuNP-OmpA1, AuNP-OmpA2, and AuNP-Pal vaccinated animals exhibited serum cross-reactivity to *B. multivorans* and to a lesser degree to *B. cenocepacia*, confirming our prediction that these antigens can elicit highly cross-reactive antibodies (Fig. 9). These findings were contrary to our expectation that endpoint titers would gradually and collectively decrease when serum was probed against the more evolutionarily distinct species. Instead, cross-reactivity was effectively a binary response, where animals either demonstrated high cross-reactivity or no cross-reactivity. While further work will be needed to confirm that these cross-reactive responses to OmpA1, OmpA2, or Pal translate to cross-protection, strategies will first need to be employed that selectively skew the immune response to the conserved, exposed regions of the protein being targeted by the animals with cross-reactive antibodies to *B. multivorans*- and *B. cenocepacia*. Strategies to skew the humoral response towards specific epitopes have been intensively researched in recent years in the influenza^67^ and HIV^68^ vaccine fields, which may provide approaches to overcome this issue.

In summary, we have characterized two subunit vaccine antigens, OmpA1 and OmpA2, capable of conferring significant protection to a lethal *Bpm* challenge and which can elicit high cross-reactive antibody titers to *Bm*, *B. multivorans*, and *B. cenocepacia*. As such, it appears feasible that a vaccine designed to protect against *Bpm* could provide some measure of protection against more distantly related *Burkholderia* species, though further optimization of the vaccine platform, cross-protection studies, and studies with human samples will be necessary moving forward. Such work could prove invaluable to cystic fibrosis patients or immunocompromised individuals who currently lack preventative options for pathogenic *Burkholderia* species.

## Methods

### In silico methodology

To screen for highly conserved *Burkholderia* antigens, we first downloaded protein FASTA files from the entire *Bpm* strain K96243 genome (RefSeq assembly GCF_000011545.1) from Ref Seq and input them into PSORTb v3.0.3 to assess subcellular localization using default settings^29^. Proteins predicted by PSORTb to be localized to the outer membrane were selected for further analysis. Additionally, proteins designated as “extracellular” or “unknown” were selected for further analysis if they were identified in prior outer membrane proteomic studies^30^. The BV-BRC proteome comparison tool (beta version) was used to compare homologous protein sequences of *Bm* strain ATCC 23344 (RefSeq assembly GCF_000011705.1), *B. cenocepacia* strain K56-2 (GenBank contig accessions reported in Supplementary Data 1), and *B. multivorans* strain ATCC 17616 (RefSeq assembly GCF_000018505.1) to the *Bpm* strain K96243 reference sequences^31^. Proteins with >90% average sequence coverage and >90% average sequence identity among the four species were selected for further analysis. Of the remaining 24 proteins, six were manually removed due to known undesirable properties of homologous proteins in other bacterial species (reasoning for removal is listed in Supplementary Data 1). The remaining proteins were analyzed with DeepTMHMM v1.0.20 to predict transmembrane topology^32^. Proteins predicted to adopt a β-barrel conformation were removed due to perceived technical challenges with recombinant expression in a native-like conformation and subsequent conjugation to the gold nanoparticle vaccine platform. The remaining 11 proteins were next input into Vaxi-DL^33^ and VaxiJen v2.0^34^ using default settings to assess the likelihood that these proteins were antigenic. Proteins predicted to be non-antigenic were removed. BLASTp^46^ and Clustal Omega^47^ were used to align the primary amino acid sequences of OmpA1, OmpA2, and Pal. ChimeraX v1.6.1^69^ was used to visualize the Clustal Omega aligned amino acid residues on AlphaFold-predicted structures (OmpA1 - AFDB accession AF-Q63W89-F1, OmpA2 - AFDB accession AF-Q63RZ9-F1, Pal - AFDB accession AF-Q63RA7-F1)^48^. GraphPad Prism v10.2.2 and BioRender were used to generate all figures.

### Bacterial strains and growth conditions

*E. coli* BL21(DE3) (New England Biolabs) and Rosetta 2(DE3) pLysS (MilliporeSigma) were used for cloning, transformation, and recombinant protein expression. Post-transformation, both strains were maintained in Luria-Bertani (LB) medium supplemented with 50 µg/mL kanamycin (Sigma-Aldrich). Rosetta 2(DE3) pLysS LB was additionally supplemented with 34 µg/mL chloramphenicol (Sigma-Aldrich). Glycerol stocks were streaked on antibiotic-supplemented LB plates and incubated at 37 °C for 18-24 h. *Bpm* strain Bp82^70^, *Bm* strain CLH001^71^, *B. multivorans* strain ATCC 17616, and *B. cenocepacia* strain K56-2 were used for whole bacteria ELISAs. *Bpm* strain K96243 was used for all challenge studies. *Burkholderia* glycerol stocks were streaked on LB agar plates (*Bpm* strain K96243, *B. multivorans*, and *B. cenocepacia*), LB plates supplemented with 100 µg/mL adenine (Sigma-Aldrich) and 5 µg/mL thiamine hydrochloride (Sigma-Aldrich) (*Bpm* strain Bp82), or LB plates supplemented with 4% glycerol (Thermo Fisher Scientific) and 200 μM FeSO_4_ (Sigma-Aldrich) (*Bm* CLH001). Plates were incubated at 37 °C for 36–48 h. Bacterial cultures were incubated at 37 °C with constant shaking at 200 rpm for 12–18 h. The OD_600_ was then measured, and bacteria were diluted in Dulbecco’s phosphate-buffered saline (PBS; Corning) to the desired concentration.

### Cloning and recombinant protein induction in *E. coli*

Genomic DNA was isolated from *Bpm* strain K96243 with a GenElute Bacterial Genomic DNA purification kit (Sigma-Aldrich) according to manufacturer's instructions. The genes of interest were PCR amplified from genomic DNA using Q5 polymerase (New England Biolabs) with High GC Enhancer buffer (New England Biolabs) according to manufacturer instructions. Amplified sequences of BPSL0999 and BPSL2765 were shortened at the 5’-end to exclude the first 22 and 21 amino acid residues, respectively, which are predicted signal peptides. For BPSL2522, additional base pairs were removed encoding the first 84 residues of the translated protein due to the presence of hydrophobic amino acids in this region. The Gibson Assembly primers used for gene amplification are reported in Supplementary Table 1 and were purchased from Integrated DNA Technologies. PCR products were purified using a PCR Cleanup Kit (Qiagen) according to manufacturer instructions. Amplified genes of interest with Gibson Assembly overhangs and an N-terminal 6X histidine tag sequence were assembled into a PCR-linearized pET-30a(+) backbone using NEB Gibson Assembly reagents and protocol, with a 2-fold molar excess of insert-to-backbone utilized. The assembled plasmid was then transformed into BL21(DE3) competent *E. coli* for the OmpA1 construct and Rosetta 2(DE3) pLysS competent *E. coli* for the OmpA2 and Pal constructs. Rosetta 2(DE3) pLysS was used for these constructs because Pal- and OmpA2-transformed BL21(DE3) exhibited significantly reduced growth rates and only produced low amounts of Pal and OmpA2. Single colonies were selected, grown up in LB supplemented with selective antibiotics, and stored at -80 °C in LB supplemented with 15% glycerol.

For protein induction, single colonies were transferred to 20 mL antibiotic-supplemented LB and incubated for 12–18 h at 37 °C with constant shaking at 200 rpm. Overnight cultures were then diluted 1:100 in fresh antibiotic-supplemented LB to a total volume of 1–2 L and incubated at 37 °C with 200 rpm shaking for 3–5 h until OD_600_ reached 0.6. Isopropyl β-D-1-thiogalactopyranoside (GoldBio) was then added to a final concentration of 1 mM to induce protein expression. Four hour post-induction, the bacteria were centrifuged into a pellet at 4000 × *g* for 10 min at 4 °C. The supernatant was discarded, and the resultant pellet was stored at −80 °C until ready for protein purification.

### Purification of recombinant proteins

Induced *E. coli* pellets were kept on ice and suspended with a magnetic stir bar in 25–50 mL of lysis buffer [50 mM Tris hydrochloride (MilliporeSigma), 500 mM sodium chloride (Thermo Fisher Scientific), 20 mM imidazole (Sigma-Aldrich), 10% (v/v) glycerol, 1% (v/v) Triton X-100 (Sigma-Aldrich), 1X cOmplete Protease Inhibitor Cocktail (Roche Life Science), 1X DNase I (Sigma-Aldrich), pH 7.5]. The bacteria were then sonicated with a 1/2” probe set to 50% amplitude in 10 s pulses with intermittent 30 s rest periods on ice. The resulting lysate was centrifuged at 22,000 × *g* for 1 h at 4 °C to pellet intact bacteria and debris. The supernatant was then filtered with a 0.2 µm PES membrane. Filtered supernatant was applied onto a 5 mL HisTrap HP column (Cytiva) equilibrated in HisTrap Buffer A (50 mM Tris hydrochloride, 500 mM sodium chloride, 20 mM imidazole, 10% (v/v) glycerol, pH 7.5) and connected to an ÄKTA pure liquid chromatography system (FPLC; Cytiva) kept at 4 °C. The column was then washed with 5 column volumes of Buffer A. Next, protein was eluted by slowly increasing the amount of HisTrap Buffer B (50 mM Tris hydrochloride, 500 mM sodium chloride, 500 mM imidazole, 10% (v/v) glycerol, pH 7.5) from 0% to 40% over the course of 15 column volumes and then from 40% to 100% in 5 column volumes. Eluent was collected in 5 mL fractions. Ten microliters each fraction was applied to a 12% SDS-PAGE gel (Bio-Rad) and stained with Imperial Protein Stain (Thermo Fisher Scientific). Fractions containing purified protein were combined and dialyzed overnight at 4 °C into PBS using dialysis cassettes with a 10 kDa molecular weight cutoff (MWCO; Thermo Fisher Scientific). Dialyzed protein was then concentrated to ≥1.25 mg/mL using an Amicon spin concentrator with 10 kDa MWCO (Sigma-Aldrich). Next, the concentrated protein was applied to a Pierce High-Capacity Endotoxin Removal Column (Thermo Fisher Scientific) for 1 h at room temperature on a tube inverter to reduce the presence of *E. coli* lipopolysaccharide. The final protein concentration was then assessed via Micro BCA Protein Assay Kit (Thermo Fisher Scientific) according to manufacturer instructions. The purified protein was run on another 12% SDS-PAGE gel and Coomassie stained to assess relative purity of the protein via ImageJ gel densitometry^72^. Protein was then diluted in PBS to a final protein concentration of 1 mg/mL and final glycerol concentration of 10% (v/v) before flash-freezing in liquid nitrogen and storing at −80 °C.

### Peptidoglycan binding assay

We measured the ability of each protein to bind to PGN using a previously described method^73^. Briefly, 50 µg of OmpA1, OmpA2, Pal, or bovine serum albumin (BSA; negative control) were mixed with 50, 100, or 200 µg of insoluble *E. coli* K12 peptidoglycan (Invivogen) in a final volume of 100 µL binding buffer [10 mM sodium phosphate (Sigma-Aldrich) and 50 mM sodium chloride, pH 7.4]. Each mixture was vortexed every 10 min and was allowed to bind for 1 h at room temperature (RT). After the incubation, tubes were centrifuged at 16,100 × *g* for 20 min at RT, and supernatant was collected. The resulting pellet was then washed three times with 500 µL binding buffer, spinning at 16,100 × *g* for 20 min after each wash. After the final wash, the pellet was reconstituted in 10 µL 4X Laemmli buffer (Bio-Rad). Five Microliters of supernatant or all the reconstituted pellets were applied to 12% SDS-PAGE gels and subsequently Coomassie-stained.

### Gold nanoparticle synthesis

Spherical gold nanoparticles were synthesized using the Turkevich method^74^. All glassware and stir bars were thoroughly cleaned with aqua regia [1 part nitric acid (Sigma-Aldrich) and 3 parts hydrochloric acid (Sigma-Aldrich)] prior to use and then rinsed with molecular grade water (Corning). All reagents were reconstituted in molecular-grade water. 90 mL of 1 mM gold (III) chloride trihydrate (Sigma-Aldrich) was first brought to a boil with vigorous stirring. A covered vessel was used to minimize volume loss from evaporation. Upon boiling, 10 mL of 90 mM sodium citrate (Sigma-Aldrich) is quickly spiked into the solution. The solution was vigorously stirred at 95–100 °C for another 15 min before turning off the heat and allowing the solution to slowly cool to room temperature. The resulting nanoparticles were then stored at 4 °C.

The concentration of unmodified AuNPs was approximated using the following equation from Haiss et al.^75^:

$$c=\frac{{A}_{450}}{{\varepsilon }_{450}}$$

where c is the concentration in moles of nanoparticles per liter, A_450_ is the measured absorbance of the stock solution of AuNPs at 450 nm with a path length of 1 cm, and ε_450_ is the extinction coefficient of 13 nm AuNPs at 450 nm, reported by the authors as 1.39 × 10^8^ L/mol-cm^75^. A_450_ was measured with an Agilent BioTek Epoch plate reader.

### Conjugation of proteins to gold nanoparticles

A 3.4 kDa heterobifunctional PEG linker with thiol and NHS functional groups (Nanocs) was reconstituted at 50 mg/mL in molecular grade water. This stock solution was always used immediately after reconstitution due to the short half-life of NHS in aqueous solutions. 50 mg/mL PEG was then added to 1 mg/mL aliquots of protein at an experimentally optimized, lot-specific linker-to-protein molar ratio ranging from 10 to 50. The PEG and protein were allowed to react for 2 h at RT on a tube inverter. After the reaction, the resulting PEGylated protein was passed through a 0.2 µm PES filter before applying it in 500 µL batches to a Superdex 75 Increase 10/300 GL size exclusion column (Cytiva) connected to an ÄKTA pure FPLC system. PBS was used as the FPLC running buffer, and 1 mL fractions were collected. Each fraction was then run on a 12% SDS-PAGE gel, which was subsequently stained using a Pierce Silver Staining kit (Thermo Fisher Scientific). Fractions containing PEGylated protein were combined and concentrated to ≥1.25 mg/mL using an Amicon Ultra Centrifugal concentrator with 10 kDa MWCO. Final concentration was determined with a Micro BCA kit. The degree of PEGylation is quantified by applying 5 µL of the PEGylated protein to a 12% SDS-PAGE gel, Coomassie staining, and calculating the relative abundance of the different bands of protein using ImageJ densitometry^72^. Finally, PEGylated proteins were diluted to a final concentration of 1 mg/mL and glycerol concentration of 10% (v/v) before being flash-frozen in liquid nitrogen and stored at −80 °C.

When ready to proceed with AuNP conjugation, 1 mg/mL PEGylated proteins were reacted with AuNPs at a v/v ratio of 1:9 for 2 h at RT on a tube inverter. For PEGylated nanoparticles lacking protein, 50 mg/mL linker was directly reacted with AuNPs at a v/v ratio of 1:9. After 2 h, the constructs were separated into 1 mL aliquots in microcentrifuge tubes and centrifuged at 16,100 × *g* for 2 h at 4 °C. Supernatants were collected to measure the amount of unreacted protein via Micro BCA to estimate the amount of protein linked to the nanoparticles. Pellets were resuspended in 1 mL of PBS and centrifuged again for a total of two washes. After the final centrifugation step, pellets are reconstituted in 100 µL of PBS, and aliquots are recombined into a single tube. Nanoparticles were then stored at 4 °C until ready for use.

### Gold nanoparticle visible light spectroscopy

The absorbance spectrum of 1:10 PBS diluted nanoparticles was measured via visible light spectroscopy on an Agilent BioTek Epoch plate reader. Absorbance was read from 450 to 700 nm, in 1 nm increments, and with a 1 cm path length. *λ*_SPR_ is reported as the wavelength at which maximum absorbance is measured. Larger or surface-functionalized nanoparticles exhibit an increase in λ_SPR_^75^. A_SPR_ is reported as the absorbance at *λ*_SPR_. Aggregation can be detected as significant absorbance in the 600–700 nm range or a decrease in the ratio of A_SPR_ to A_450_.

### Zetasizer measurements

Particle size distribution of the 1:10 PBS diluted AuNP constructs was measured on a Malvern Zetasizer µV equipped with an 830 nm laser and detector angle of 90°. Refractive index and absorption settings were 0.200 and 3.320, respectively. Two µL of nanoparticles were applied to a ZMV1002 quartz cuvette and measured at 25 °C. Samples were measured a total of three times, and each measurement consisted of 10 runs lasting 10 s each. Zeta potential was measured using laser Doppler velocimetry with mixed mode measurement and phase analysis light scattering, using a Malvern Zetasizer Nano ZS equipped with a 633 nm laser and 12.8° scattering beam angle. Nanoparticle zeta potential was determined in 0.1X PBS or 9 mM sodium citrate buffer. Samples were quantified using the Smoluchowski approximation and measured three to eight times to estimate error; each independent measure consisted of 20 runs.

### Transmission electron microscopy

Unmodified AuNPs were directly applied as a 5 µL droplet to Formvar/Carbon 200 Mesh, Cu grids (Electron Microscopy Sciences) for 5 min. Remaining liquid was carefully removed with a wedge of filter paper and then dried under a lamp. For the conjugated AuNP constructs, 1:10 PBS diluted nanoparticles were applied to the grids as above and then stained with 2% aqueous uranyl acetate for 1 min. Grids were imaged on a JEOL JEM-1400 transmission electron microscope at 80 kV.

### Agarose gel electrophoresis of conjugated gold nanoparticles

Thirty µL of 1:10 PBS diluted nanoparticles were directly applied to the center of a 0.5% agarose gel and ran at 100 V for ~1 h. 1X Tris-borate-EDTA buffer pH 8.3 (Bio-Rad) was used as the running buffer. Gels were directly imaged without the need of a stain due to the red coloration of the AuNPs.

### Mass spectrometry absolute quantification of gold nanoparticle-conjugated protein

We measured the concentration of AuNP-conjugated protein based on a previously established method^76^. Twenty-five microliters of AuNP-OmpA1, AuNP-OmpA2, or AuNP-Pal were mixed with 25 µL of 50 mM triethylammonium bicarbonate buffer pH 8.5 (Sigma-Aldrich). Samples were then reduced by adding Tris-(2-carboxyethyl)-phosphine (Thermo Fisher Scientific) to the solution at a final concentration of 10 mM and incubated at 37 °C for 15 min. The samples were then cooled to RT, alkylated with 1 µL of 500 mM iodoacetamide (Sigma-Aldrich), and allowed to react for 15 min at RT in the dark. Thirty µL of 20 ng/µL trypsin (Promega) was added and incubated at 37 °C overnight. The resulting solution was centrifuged at 20,000 × *g* for 1 h to separate the AuNPs from the digested peptide fragments. Peptides were desalted using 10–200 µL HyperSep C18 SpinTips (Thermo Fisher Scientific). Peptides were dried in a Jouan RC10.22 Vacuum Concentrator and suspended in 9 µL of 1.67% acetonitrile (Thermo Fisher Scientific), 0.08% formic acid (Thermo Fisher Scientific), 0.83% acetic acid (Sigma-Aldrich), 97.42% water. AQUA Ultimate Grade heavy-isotope labeled peptides (Thermo Fisher Scientific) matching sequences found in OmpA1 (AQSVVNALAQ-R*; * = labeled with ^13^C and ^15^N), OmpA2 (VEVEVVGTQEVQ-K*; * = labeled with ^2^H), and Pal (GTSEYNLALGQ-K*; * = labeled with ^2^H) were diluted to 500 fmol/µL with 1.67% acetonitrile, 0.08% formic acid, 0.83% acetic acid, 97.42% water and 1 µL was added to the peptide cocktail from each sample for a final heavy-peptide concentration of 50 fmol/µL. The resultant 10 µL mixture was placed in an autosampler vial.

Peptide mixtures were analyzed by nanoflow liquid chromatography-tandem mass spectrometry (nanoLC-MS/MS) using a Dionex UltiMate 3000 RSLCnano, coupled online to a Thermo Orbitrap Fusion mass spectrometer (Thermo Fisher Scientific) through a Nanospray Ion Source (Thermo Fisher Scientific). A trap and elute method was used. The trap column is an Acclaim C18 PepMap100 (100 µm × 2 cm, 5 µm particle size; Thermo Fisher Scientific). The analytical column is an Acclaim C18 PepMap 100 (75 µm × 25 cm, 3 µm particle size; Thermo Fisher Scientific). After equilibrating the column in 98% solvent A (0.1% formic acid in water) and 2% solvent B (0.1% formic acid in acetonitrile), 1 µL of sample was injected onto the trap column and subsequently eluted by gradient elution onto the C18 column as follows: isocratic at 2% B, 0–4 min (500 nL/min); isocratic at 2% B, 4–5 min (decrease flow to 300 nL/min); 2 to 4% B, 5–6 min; 4 to 28% B, 6-30 min; 28 to 42% B, 30–33 min; 42 to 90% B, 33–35 min; isocratic at 90%, 35–36 min; 90 to 4% B, 36–36.5 min; isocratic at 4% B, 36.5–37 min (increase flow to 500 nL/min); 4 to 90% B, 37–38.5 min; isocratic at 90% B; 38.5–39 min; 90 to 2% B, 39–40 min; and isocratic at 2% B till 45 min.

All LC-MS/MS data were acquired using XCalibur v4.4 (Thermo Fisher Scientific) in positive ion mode using a targeted data-dependent acquisition method. The survey scans (*m*/z 350–2000) were acquired in the Orbitrap at 60,000 resolution (*m*/*z* = 400) in profile mode, with maximum injection time set to auto and an AGC target set to standard. The S-lens RF level was set to 60. Isolation for targeted MS2 scans is performed in the quadrupole with a 1.6 Da isolation window, and HCD MS/MS acquisition is performed in centroid mode with detection in the Orbitrap at 15,000 resolution with the following settings: normalized collision energy 28%; maximum injection time set to dynamic with an input desired minimum points across the peak set to 6; AGC target set to standard. The target mass lists for the two experiments are provided in Supplementary Table 2.

Skyline v22.2.0.351 was used for targeted analysis. Single-point quantitation was performed on the sample showing the highest similarity in area under the curve (AUC) between the heavy-labeled and light peptides. The summed MS2 AUC of the light and heavy peptides was used to calculate the molar concentrations (fmol/µL). Quantitation for the remaining samples was derived from the ratio of the light peptide areas. The approximate number of conjugated proteins per nanoparticle was determined by dividing the mass spectrometry-measured molar concentration of conjugated protein by the mathematically approximated^75^ molar concentration of nanoparticles.

### Animal vaccination and challenge studies

Six-to-8-week-old female C57BL/6 mice were purchased from Charles River Laboratories (Wilmington, MA) and housed in microisolator cages under pathogen-free conditions. Rodent chow and water were provided *ad libitum*, and a 12 h light cycle was maintained. All procedures were reviewed and approved by the Institutional Animal Care and Use Committee of the University of Texas Medical Branch (protocol 0503014 F). Animals were allowed to acclimate for 1 week prior to conducting experiments.

Mice were lightly sedated with isoflurane and immunized i.n. with 50 µL (25 µL per nare) of PBS diluted AuNP-OmpA1, AuNP-OmpA2, or AuNP-Pal every two weeks for a total of three doses. Negative control animals were either given PBS (saline control) or PEGylated AuNPs without an antigen plus 20 µg VacciGrade CpG ODN 2395 (Invivogen) (adjuvant control). Low-dose vaccines (*N* = 10) consisted of ~1.5 µg conjugated protein and exactly 20 µg of VacciGrade CpG ODN 2395, whereas high-dose vaccines (*N* = 8) contained approximately 6 µg of conjugated protein and 20 µg of VacciGrade CpG ODN 2395. Adjuvant control animals were given a concentration of nanoparticles equivalent to the highest nanoparticle concentration of the other groups, as determined by measuring the OD_450_. Two weeks after the final immunization, animals were anesthetized with isoflurane and bled retro-orbitally to assess circulating antibody responses. Blood was collected into Microvette tubes and was allowed to clot at RT for 30 min. Blood was then centrifuged at 2000 × *g* for 10 min at 4 °C, and serum was collected and stored at −80 °C. Three weeks after receiving their full immunization regimen, animals were transferred to an ABSL3 facility and challenged under isoflurane sedation i.n. with 50 µL (25 µL per nare) of either 2.2 × LD_50_ (low dose vaccination study) or 5.0 × LD_50_ (high dose vaccination study) of *Bpm* strain K96243 in PBS. Animals were weighed and checked daily, or twice daily when weight loss surpassed 10%. Clinical scores were assigned as follows: 1 = active and healthy appearance; 2 = mild lethargy; 3 = ruffled fur, hunched posture, and mild lethargy; 4 = ruffled fur, hunched posture, limited mobility; 5 = moribund. Animals that reached 20% weight loss or a clinical score of 5 were humanely euthanized. After 21 days, remaining animals were euthanized via CO_2_ asphyxiation and cervical dislocation, and lungs, livers, and spleens of surviving mice were collected. Tissues were then weighed and mechanically disrupted with Fisherbrand disposable tissue grinders. 1:10 PBS dilutions of the homogenized tissues were plated on Ashdown agar plates to measure tissue colonization. The remaining 90% volume of the initial tissue suspension was plated on a large Ashdown plate to confirm sterility. Ashdown plates were cultured in a 37 °C incubator for 60–72 h before counting colonies.

To assess T cell responses and secreted IgA responses, a separate cohort of animals (*N* = 5) was given the low-dose immunization regimen as described and were humanely euthanized 10 days after the final immunization via CO_2_ asphyxiation. BAL fluid was collected by administering 1 mL PBS to the lungs through a small incision in the trachea and then aspirating the fluid. BAL was kept on ice and subsequently stored at −80 °C. NALTs were collected according to a previously published protocol^77^. In brief, upper palates were decontaminated with 70% ethanol and then excised with a no. 11 surgical blade. The palate was then gently peeled back with forceps in one piece, and the palate was subsequently washed eight times with 250 µL complete RPMI 1640 (Thermo Fisher Scientific) [cRPMI; 10% heat-inactivated fetal bovine serum (FBS; Thermo Fisher Scientific), 100 units/mL penicillin (Thermo Fisher Scientific), 100 µg/mL streptomycin (Thermo Fisher Scientific), 1X Glutamax (Thermo Fisher Scientific), 1X non-essential amino acids (Thermo Fisher Scientific), 1 mM sodium pyruvate (Thermo Fisher Scientific), and 50 µM cell culture grade β-mercaptoethanol (Sigma-Aldrich)] in a 48 well plate. NALTs were then transferred to new, sterile 48 well plates and placed in a humidified 37 °C + 5% CO_2_ incubator. Half of the NALT supernatants were collected and replenished with fresh cRPMI every 24 h for three days, and supernatants were stored at −80 °C. Spleens were collected in 5 mL PBS + 2% FBS and kept on ice. Spleens were then homogenized on a 70 µm nylon cell strainer using a syringe plunger, and the filters were washed with 10 mL of PBS + 2% FBS. Splenocytes were then centrifuged at 300 × *g* for 10 min at 4 °C. Supernatants were discarded, and cell pellets were reconstituted in 5 mL ACK lysing buffer (Thermo Fisher Scientific) for 5 min before stopping lysis with 20 mL PBS. After another centrifugation step, splenocyte pellets were resuspended in 15 mL cRPMI. Splenocytes were then counted via trypan blue (Thermo Fisher Scientific) staining on a hemacytometer. Splenocytes were centrifuged again and resuspended at ≥10^7^ cells/mL in freezing media [90% FBS and 10% cell culture grade dimethyl sulfoxide (Sigma-Aldrich)] and placed at −80 °C overnight before then being transferred to liquid nitrogen for long-term storage.

For the AuNP-OmpA2 dosing studies, animals (*N* = 4) were immunized as described with 20 µg of VacciGrade CpG ODN 2395 and ~3, 1.5, or 0.75 µg of AuNP-conjugated protein per dose. Negative control animals were administered PBS. Blood was collected via retro-orbital bleed 10 days after the second immunization and again 14 days after the third immunization via cardiac puncture. Spleens were also collected 14 days after the third immunization. Spleens and blood were processed as described above.

### ELISAs

For antigen-specific IgG, IgG1, IgG2c, and IgA ELISAs, proteins were dispensed into wells of high-binding 96-well plates at 2 µg/mL in 100 µL PBS and were incubated overnight at 4 °C. The next day, plates were washed four times with 350 µL/well of PBS + 0.1% Tween 20 (Sigma-Aldrich). PBS + 1% BSA (m/v; Sigma-Aldrich) was then added to the plates at 200 µL/well for 2 h at RT. After another four washes, serial dilutions of serum or 1:5 diluted BAL or 1:15 diluted NALT culture supernatant were added to the plate in 100 µL/well for 1.5 h at RT. After another four washes, 1:5000 diluted secondary antibody (goat anti-mouse IgG-HRP, goat anti-mouse IgG1-HRP, goat anti-mouse IgG2c-HRP, goat anti-mouse IgA-HRP; Southern Biotech) was added at 100 µL/well for 1.5 h at RT. Plates were again washed four times before adding 100 µL/well of 3,3′,5,5′-tetramethylbenzidine (TMB) substrate (SeraCare) for 5 min at RT. Then, 100 µL/well 0.18 M sulfuric acid (Sigma-Aldrich) was added to stop the reaction. Absorbance at 450 and 650 nm was measured. The absorbance measured at the 650 nm reference wavelength was subtracted from the absorbance measured at 450 nm. Endpoint titers were calculated as the highest dilution at which corrected absorbance values were higher than the equivalently diluted naïve serum average signal plus three standard deviations.

The whole bacteria ELISA protocol was based on previously published methods^78^. First, 20 mL cultures of *Bpm* strain Bp82, *Bm* strain CLH001, *B. multivorans* strain ATCC 17616, and *B. cenocepacia* strain K56-2 were incubated at 37 °C with constant shaking at 200 rpm to stationary phase (12–18 h). The bacteria were then centrifuged at 3200 × *g* for 10 min, and the supernatant was decanted. The pellet was washed with 40 mL PBS and then resuspended to an OD_600_ of 0.5 in PBS, and 100 µL/well of the live bacteria was plated onto high-binding 96-well plates. Plates were allowed to dry completely at 37 °C. From there, all steps were performed identically to the recombinant protein ELISAs, except Tween 20 concentration was reduced to 0.025% and the TMB incubation time was increased from 5 to 30 min.

### T cell recall assay

Live splenocytes (2 × 10^5^) were dispensed into wells of 96-well tissue culture-treated plates or pre-coated 96-well ELISpot plates (Mouse IFNγ, Mouse IL-17, Mouse IL-4; R&D Systems). Splenocytes from the immunized animals were stimulated with 20 µg/mL of the recombinant protein they were immunized with (OmpA1, OmpA2, or Pal) in 100 µL of cRPMI. Splenocytes from the adjuvant control animals were stimulated with each of the proteins separately to assess non-specific activation. Unstimulated control wells contained cRPMI supplemented with 2% (v/v) PBS, equivalent to the amount of PBS in the antigen-stimulated wells. Positive control wells received 1X cell stimulation cocktail (Thermo Fisher Scientific). Plates were placed in a humidified incubator at 37 °C and 5% CO_2_ for an assay-specific incubation time.

### Flow cytometry

For surface marker and intracellular cytokine staining, splenocytes were recalled with antigen for 19 h before adding brefeldin A (Thermo Fisher Scientific) and monensin (Thermo Fisher Scientific) to a final concentration of 1X. After stimulation for 24 h, five technical replicates of each stimulated or unstimulated experimental sample were combined into a single well of a new 96-well plate. Plates containing the cells were centrifuged at 300 × *g* for 10 min at 4 °C and supernatant was decanted. Plates were then washed with 200 µL/well PBS, centrifuged again, and decanted. Five µg/mL anti-mouse CD16/32 (BioLegend) was added to the wells for 10 min at 4 °C. The plates were then centrifuged and decanted, before adding 100 µL/well of surface staining cocktail for 30 min at 4 °C. This cocktail consisted of experimentally titrated concentrations of anti-mouse CD3e-BUV395 (BD Biosciences; clone 145-2C11), anti-mouse CD44-BUV805 (BD Biosciences; clone IM7), anti-mouse CD4-BV510 (BioLegend; clone RM4-5), anti-mouse/human CD45R/B220-BV785 (BioLegend; clone RA3-6B2), anti-mouse CD8a-PerCP/Cy5.5 (Thermo Fisher Scientific; clone 53-6.7), anti-mouse CD62L-PE/Cy7 (BioLegend; clone MEL-14), and Zombie NIR fixable viability dye (BioLegend) diluted in FACS buffer (BioLegend). We included unstained, single-stained, and fluorescence minus one (FMO) controls. The plate was then washed again with PBS before adding 100 µL/well fixation buffer (BioLegend) for 20 min at 4 °C. Plates were washed twice with 1X permeabilization wash buffer (BioLegend) before incubating them overnight at 4 °C with 100 µL/well intracellular cytokine staining cocktail. This cocktail consisted of experimentally titrated amounts of anti-mouse IL-2-BV421 (BioLegend; clone JES6-5H4), anti-mouse IL-17A-BV711 (BioLegend; clone TC11-18H10.1), anti-mouse IL-4-PE (BioLegend; clone 11B11), and anti-mouse IFNγ-APC (BioLegend; clone XMG1.2) diluted in 1X permeabilization wash buffer. The following morning, plates were washed with 1X permeabilization wash buffer before finally resuspending the pellets in 250 µL/well of FACS buffer. The samples were then immediately run on a BD FACSymphony A5 SE. All data analysis was performed with FlowJo v10.10.

To measure secreted cytokines, splenocytes were recalled with antigen for 48 h, and half the supernatant was collected and replenished at the 24 and 48 h time points. Supernatants were stored at −80 °C. Thawed, undiluted supernatants were analyzed using a LEGENDplex Mouse Th (12-plex) kit (Biolegend) according to manufacturer instructions. Reported IFNγ concentrations were from the 24 h stimulation time point, and IL-17A concentrations were reported from the 48 h time point. Samples were analyzed with a BD FACSymphony A5 SE. Data analysis was performed with FlowJo v10.10.

### ELISpots

The ELISpot plates were processed according to manufacturer instructions. IFNγ ELISpot plates were processed after 24 h of antigen recall, while the IL-17A and IL-4 ELISpots were processed after 48 h of stimulation. Plates were imaged and counted with a CTL ImmunoSpot S6 Universal M2 ELISpot Reader.

### Statistical analysis

Statistical analysis was performed with GraphPad Prism v10.2.2. Bacteria-specific IgG endpoint titers were compared via Kruskal-Wallis with Dunn’s multiple comparison test. BAL and NALT IgA ELISA absorbance values were compared via two-tailed, unpaired Student’s *t*-tests. In the AuNP-OmpA2 dosing study, OmpA2-specific IgG endpoint titers were log transformed and compared using a matched pairs two-way ANOVA with Šidák correction. Total splenic lymphocyte counts and proportions of CD44/CD62L-expressing T cells were compared via one-way ANOVAs with Dunnett’s multiple comparison correction. Mouse body weights and LEGENDplex-measured cytokine concentrations were compared via one-way ANOVAs with Tukey multiple comparisons correction. The proportions of IFNγ- or IL-17A-secreting cells as measured by ELISpot or flow cytometry were compared via matched-pairs two-way ANOVAs with Fisher’s LSD tests, except in the AuNP-OmpA2 dosing study in which a one-way ANOVA with Tukey post hoc was used instead. Kaplan-Meier survival curves of the vaccinated groups were compared to the saline control (low dose vaccination study) and adjuvant control (high dose vaccination study) via log-rank tests, and adjusted p values were calculated using the Bonferroni multiple comparison correction. Organ weights normalized to total body weight were compared by unpaired, two-tailed Student’s *t*-tests. Graphs depicting endpoint titers are displayed as the geometric mean ± geometric SD. All other graphs display mean ± SD. A *p* value < 0.05 was considered statistically significant.

## Supplementary information

Supplementary Information

Supplementary Data 1

## Data Availability

The entire reverse vaccinology dataset is included in the manuscript as Supplementary Data 1. The raw data files used to generate figures and any newly created materials will be made available upon request from the corresponding author.
