# Supplementary material for: Evaluation of highly conserved Burkholderia pseudomallei outer membrane proteins as protective antigens against respiratory melioidosis
Source: NPJ Vaccines. 2025 Aug 6;10:186. doi: 10.1038/s41541-025-01246-2 (PMC12328632; doi:10.1038/s41541-025-01246-2)
Supplement: Supplementary file 1 — Supplementary Information [file 41541_2025_1246_MOESM1_ESM.pdf]

| Score | Expect | Method | Identities | Positives | Gaps |
|---|---|---|---|---|---|
| 61.6 bits(148) | 2e-16 | Compositional matrix adjust. | 41/128(32%) | 63/128(49%) | 10/128(7%) |

```
OmpA1  97   SLKLNVPSSVTFATDQYAITPAFTPLLNDLATTLN-QNPQITASVVGYTDSTGSAAHNQT  155
            S K+   +   F  D+  + PA    L++LA  +   N ++  +  GYTD  GS  +N
OmpA2  91   SQKITYQADTLFDFDKAVLKPAGKQKLDELAAKIQGMNVEVVVAT-GYTDRIGSDKYNDR  149

OmpA1  156  LSQNRAQSVVNALAQRGVAANRLSAQGMGASNPIADN-ATEAGRAQ-------NRRVEIY  207
            LS  RAQ+V + L  +GV AN++  +G G  NP+  N   +  R Q       +RRVE+
OmpA2  150  LSLRRAQAVKSYLVSKGVPANKVYTEGKGKRNPVTGNTCKQKNRKQLIACLAPDRRVEVE  209

OmpA1  208  LRAPQAAQ  215
            +   Q  Q
OmpA2  210  VVGTQEVQ  217
```

| Score | Expect | Method | Identities | Positives | Gaps |
|---|---|---|---|---|---|
| 64.7 bits(156) | 5e-18 | Compositional matrix adjust. | 43/124(35%) | 61/124(49%) | 11/124(8%) |

```
OmpA1  89   QVTEQPDGSLKLNVPSS------VTFATDQYAITPAFTPLLNDLATTLNQNPQITASVVG  142
            QVT  P     LN P+S      V F  D Y++   +  LL   A  L  +PQ    + G
Pal    48   QVTVDP-----LNDPNSPLAKRSVYFDFDSYSVQDQYQALLQQHAQYLKSHPQRHILIQG  102

OmpA1  143  YTDSTGSAAHNQTLSQNRAQSVVNALAQRGVAANRLSAQGMGASNPIADNATEAGRAQNR  202
             TD  G++ +N  L Q RA++V  AL+  GV   ++ A  +G   P+A    EA  AQNR
Pal    103  NTDERGTSEYNLALGQKRAEAVRRALSLLGVGDAQMEAVSLGKEKPVALGHDEASWAQNR  162

OmpA1  203  RVEI  206
            R ++
Pal    163  RADL  166
```

| Score | Expect | Method | Identities | Positives | Gaps |
|---|---|---|---|---|---|
| 36.6 bits(83) | 6e-08 | Compositional matrix adjust. | 24/82(29%) | 39/82(47%) | 0/82(0%) |

```
OmpA2  102  FDFDKAVLKPAGKQKLDELAAKIQGMNVEVVVATGYTDRIGSDKYNDRLSLRRAQAVKSY  161
            FDFD   ++   +  L + A  ++      ++  G TD  G+ +YN  L  +RA+AV+
Pal    68   FDFDSYSVQDQYQALLQQHAQYLKSHPQRHILIQGNTDERGTSEYNLALGQKRAEAVRRA  127

OmpA2  162  LVSKGVPANKVYTEGKGKRNPV  183
            L   GV   ++     GK  PV
Pal    128  LSLLGVGDAQMEAVSLGKEKPV  149
```

**Fig. S1. BLASTp Pairwise Comparisons of OmpA1, OmpA2, and Pal.** Protein sequence FASTA files of OmpA1 (BPSL0999), OmpA2 (BPSL2522), and Pal (BPSL2765) from *Bpm* strain K96243 were obtained from UniProt. Pairwise comparisons of the sequences were performed with the NCBI standard protein BLAST web tool with default parameters.

```
OmpA2      -MNKLSKLAFI---AATAVMAASASAQSVPASR---------------QAVNDNWVNGTG 41
OmpA1      -MNTKIATRLSVFALAGALLAGCATQQGTNTAVGTGTGAALGAGIGALAGGGKGAAIGAG 59
Pal        MMSKKLRLAFAM--LMIGALAACKSGVKLD----------------EHA--NQGDA---- 36
            *..     :       . :*.. :                          ... .

OmpA2      EWVWMNGTNELCWRDAFWTPATANAKCDGALVAQAPAPAPVAPVAPAITSQKITYQADTL 101
OmpA1      VGALVGGVTGYNWQAIKN-KLAPSAQQTGTQVTEQPD-----------GSLKLNVPSSVT 107
Pal        ---------------VST---QPNPEN-VAQVTVDPL----------NDPNSPLAKRSVY 67
                                  . :   : *:  *               .     ..

OmpA2      FDFDKAVLKPAGKQKLDELAAKIQGMNVEVVVATGYTDRIGSDKYNDRLSLRRAQAVKSY 161
OmpA1      FATDQYAITPAFTPLLNDLATTLNQNPQITASVVGYTDSTGSAAHNQTLSQNRAQSVVNA 167
Pal        FDFDSYSVQDQYQALLQQHAQYLKSHPQRHILIQGNTDERGTSEYNLALGQKRAEAVRRA 127
           *  *.  :       *:: *  ::          * **  *:  :*  *. .**::*

OmpA2      LVSKGVPANKVYTEGKGKRNPVTGNTCKQKNRKQLIACLAPDRRVEVEVVGTQEVQKTTV 221
OmpA1      LAQRGVAANRLSAQGMGASNPIADNATEAG--------RAQNRRVEIYLRAPQAAQ---- 215
Pal        LSLLGVGDAQMEAVSLGKEKPVALGHDEAS--------WAQNRRADLVYQQ--------- 170
           *   **   :: : . *  :*:: .  :           * :**.::

OmpA2      PAQ  224
OmpA1      ---  215
Pal        ---  170
```

**Fig. S2. Clustal Omega Multiple Alignment of OmpA1, OmpA2, and Pal.** Protein sequence FASTA files of OmpA1 (BPSL0999), OmpA2 (BPSL2522), and Pal (BPSL2765) from *Bpm* K96243 were obtained from UniProt. Multiple sequence alignment was performed with Clustal Omega. An asterisk (*****) denotes a residue that is strictly conserved. A colon (**:**) indicates the residues have highly similar physiochemical properties. A period (**.**) indicates that residues have weakly similar properties. Red arrows were overlaid to indicate residues previously identified as being involved in peptidoglycan binding and that are broadly conserved across Gram-negative species.

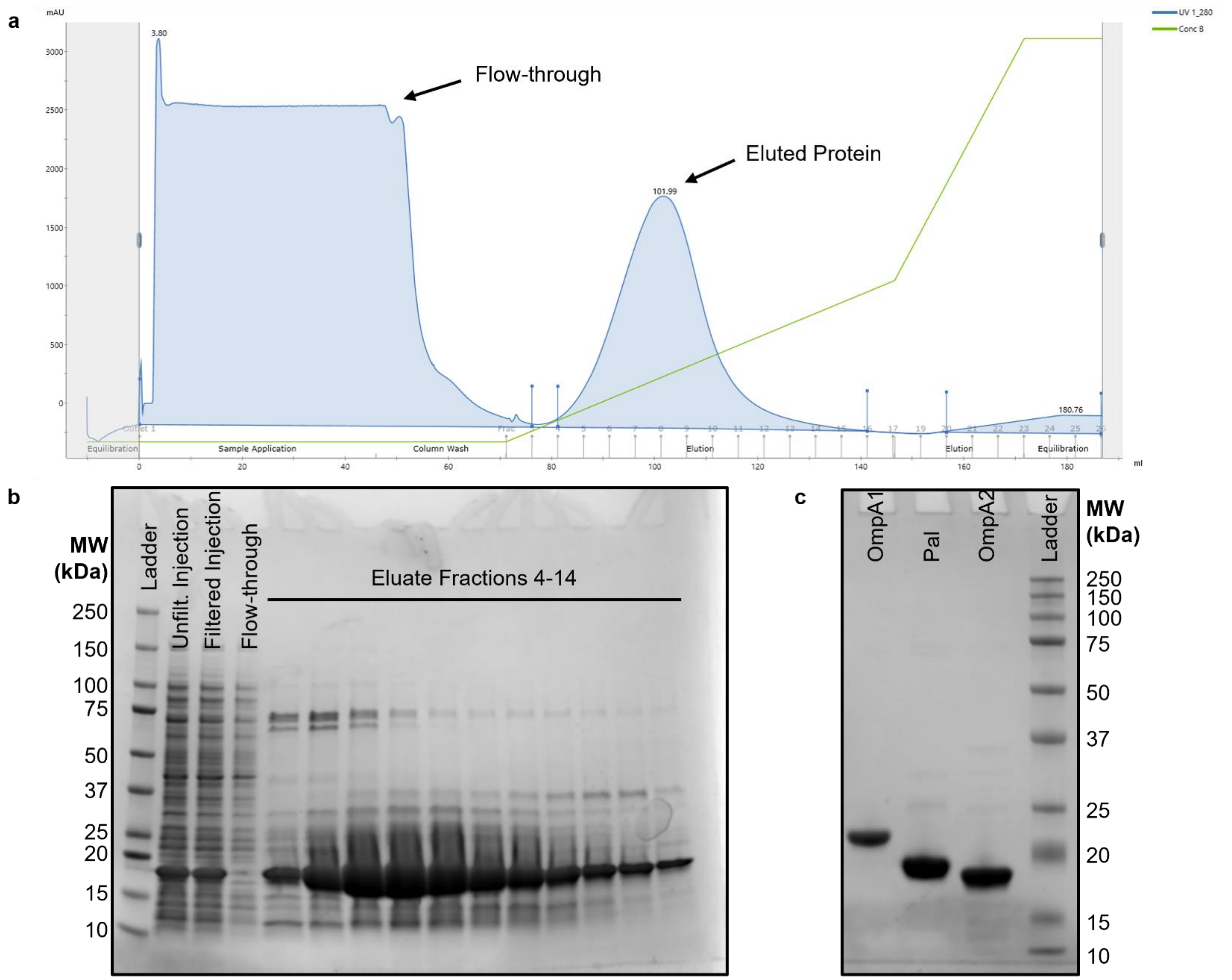


**Fig. S3. Purification of Recombinant OmpA1, OmpA2, and Pal.** *E. coli* harboring plasmids encoding OmpA1, OmpA2, and Pal were induced, pelleted, and lysed as described. Lysate was applied to a Cytiva HisTrap HP column connected to an ÄKTA pure protein purification system. **(a)** Representative chromatogram from the purification of OmpA2. **(b)** SDS-PAGE with Coomassie stain of the OmpA2-containing lysate (pre- and post-filtering), flow-through, and fractions 4 through 14 of the HisTrap HP column eluate containing OmpA2. **(c)** SDS-PAGE with Coomassie stain of fully purified OmpA1, OmpA2, and Pal. ImageJ densitometry indicates a purity of > 95%. Expected molecular weights: OmpA1 = 20.2 kDa, Pal = 17.5 kDa, OmpA2 = 16.3 kDa.

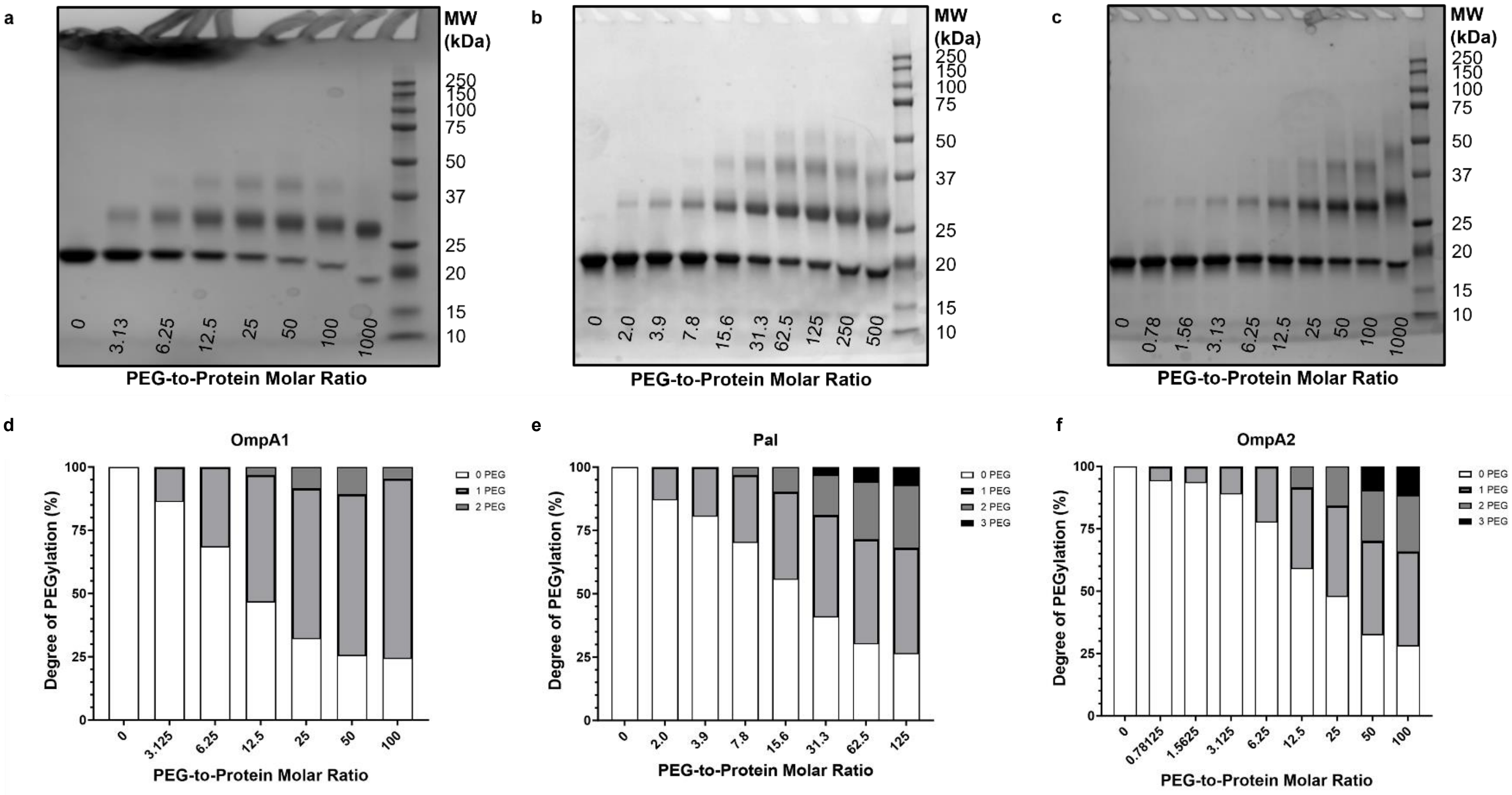


**Fig. S4. Optimization of Protein PEGylation.** Aliquots of 1 mg/mL protein were reacted with the indicated molar ratio of 3.4 kDa SH-PEG-NHS linker. SDS-PAGE with Coomassie stain of PEGylated OmpA1 **(a)**, Pal **(b)**, and OmpA2 **(c)**. ImageJ gel densitometric analysis of PEGylated OmpA1 **(d)**, Pal **(e)**, and OmpA2 **(f)**. Expected molecular weights of non-PEGylated proteins: OmpA1 = 20.2 kDa, Pal = 17.5 kDa, OmpA2 = 16.3 kDa. Graphs made with GraphPad Prism.

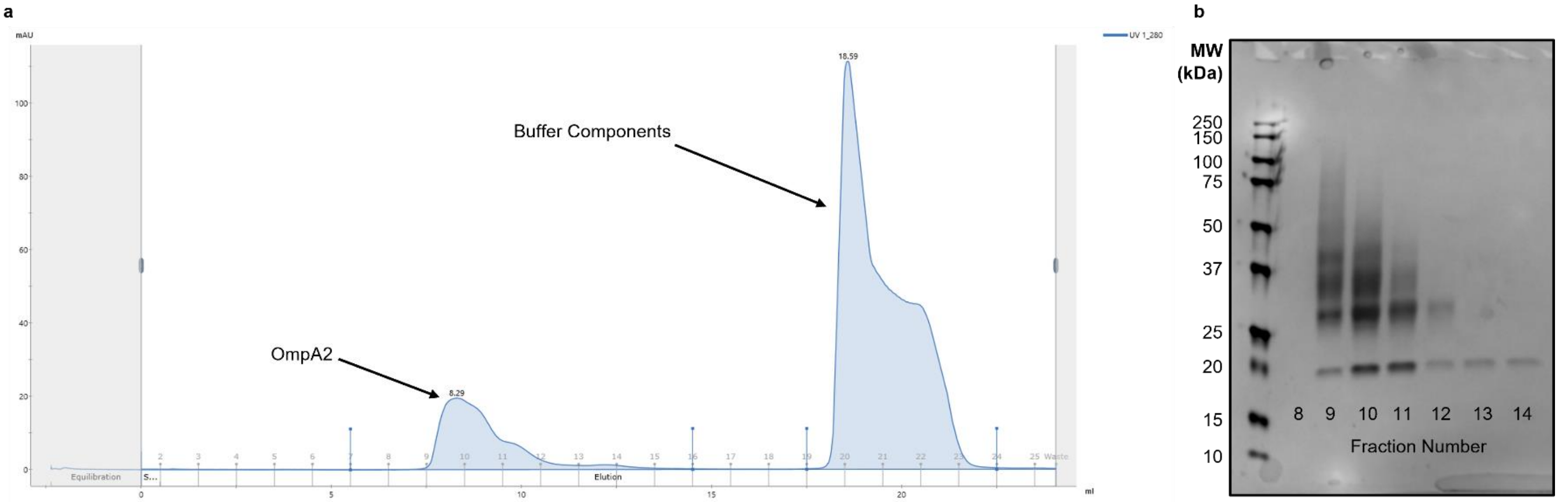


**Fig. S5. Size Exclusion Liquid Chromatography of PEGylated Proteins.** PEGylated proteins were applied to a Cytiva Superdex 75 Increase 10/300 GL column connected to an ÄKTA pure protein purification system. **(a)** Representative chromatogram from purification of PEGylated OmpA2. The peak labelled “buffer components” is devoid of protein and is thought to contain the NHS leaving group, which absorbs strongly at 280 nm, as well as unreacted linker. **(b)** Representative SDS-PAGE with silver stain of chromatography fractions 8 through 14. The expected molecular weight of non-PEGylated OmpA2 is 16.3 kDa.

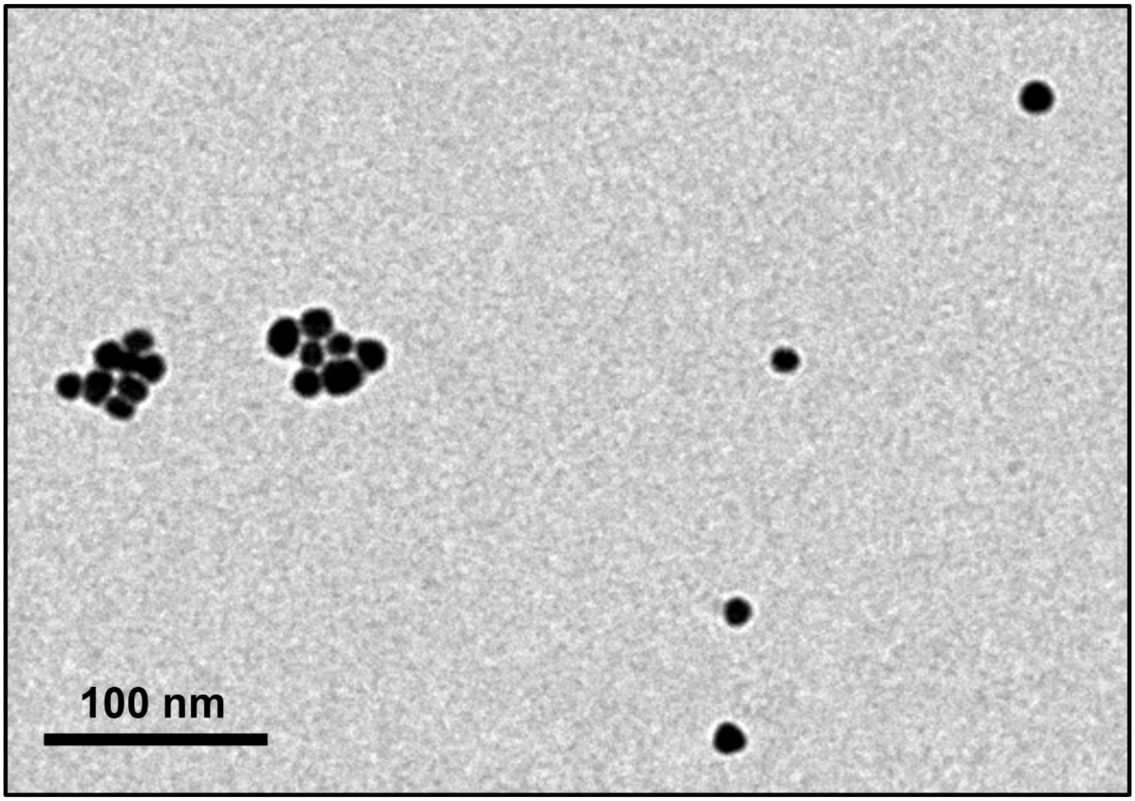


**Fig. S6. Transmission Electron Microscopy of Unmodified AuNPs.** AuNPs were directly applied as a droplet to Formvar/Carbon 200 Mesh, Cu grids and imaged on a JEOL JEM-1400 transmission electron microscope.

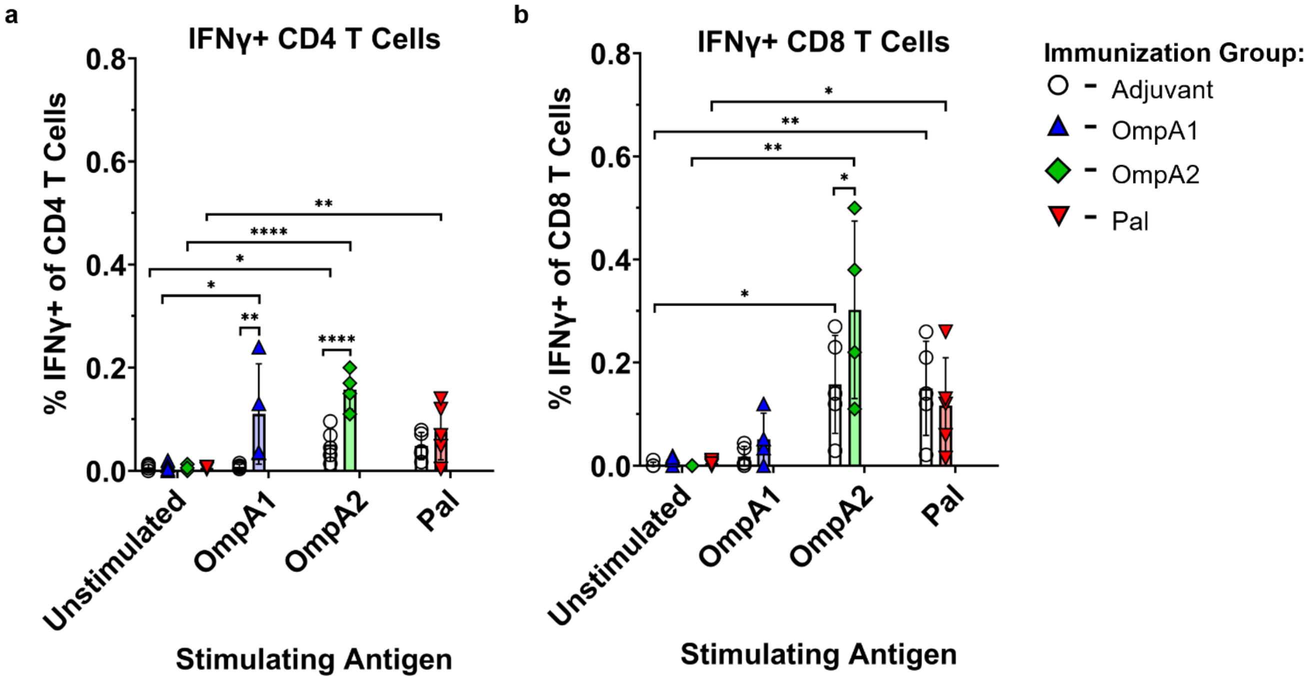


**Fig. S7. T Cell Recall Intracellular IFNγ Staining.** Splenocytes from immunized animals were stimulated with the indicated recombinant protein for 24 h, stained with fluorescent antibodies, and analyzed via flow cytometry. Intracellular IFNγ staining of CD4 **(A)** and CD8 **(B)** T cells. Groups were compared via matched-pairs two-way ANOVAs with Fisher's LSD tests. (*) $p < 0.05$, (**) $p < 0.01$, (****) $p < 0.0001$. Graphs made in GraphPad Prism.

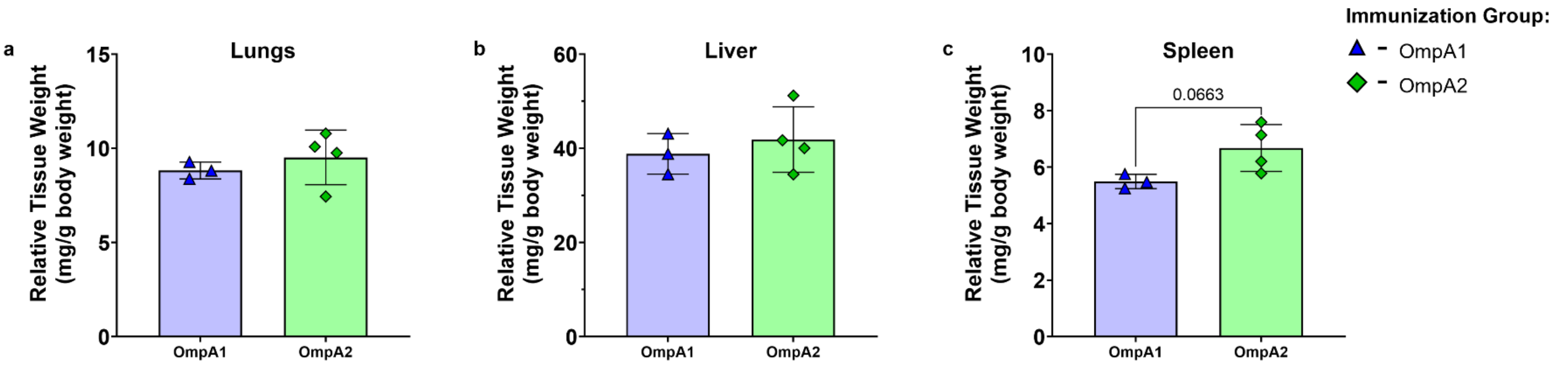


**Fig. S8. Tissue Weights Post-Infection.** Lungs **(A)**, livers **(B)**, and spleens **(C)** were collected at the challenge study endpoint and weighed. Tissue weights were normalized to body weight at time of collection. Groups were compared by unpaired, two-tailed Student's t-tests.

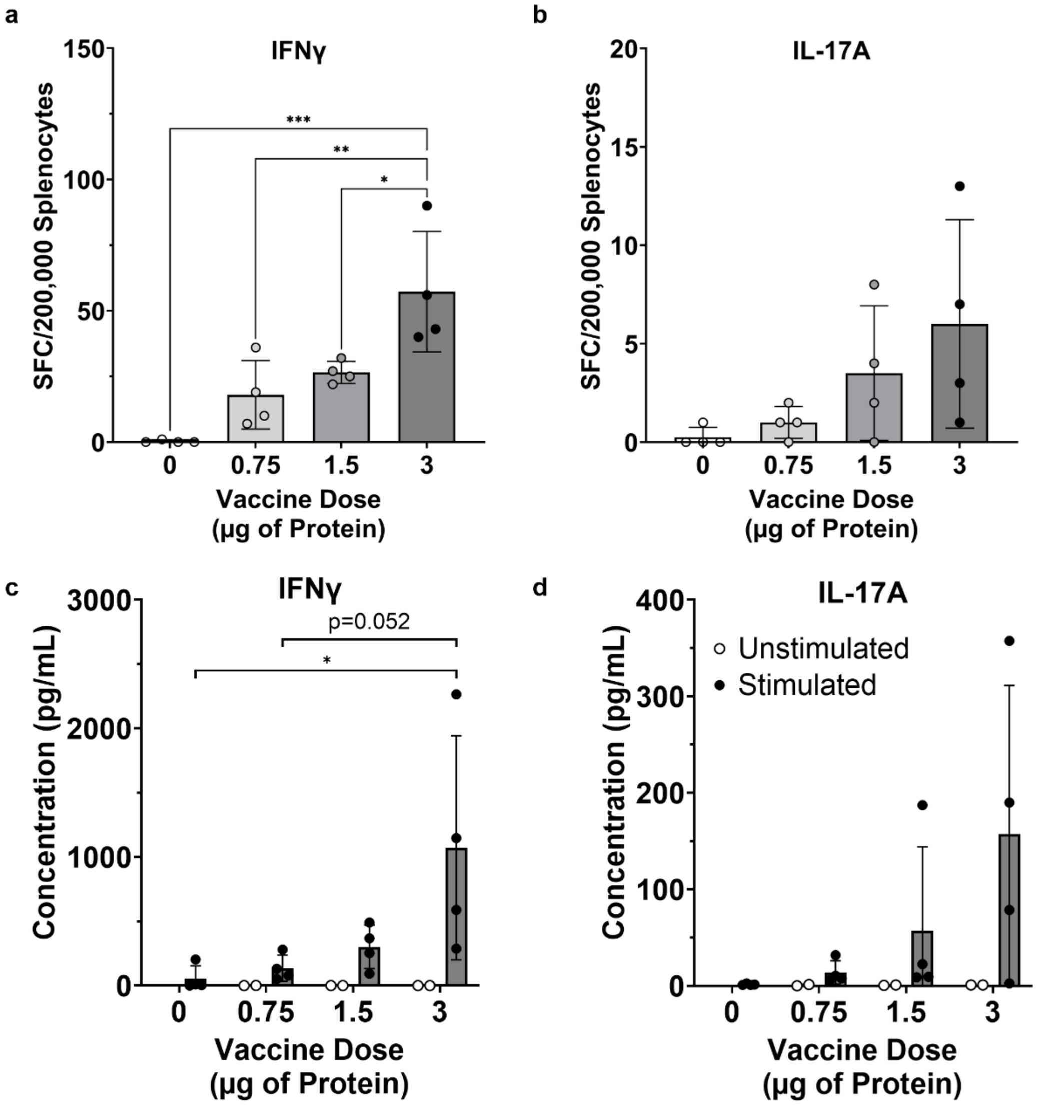


**Fig. S9. AuNP-OmpA2 Dosing Study T Cell Recall.** Mice were immunized with either 0.75, 1.5, or 3 µg/dose of OmpA2 conjugated to AuNPs and adjuvanted with VacciGrade CpG ODN 2395. At the endpoint of the study (d38), spleens were collected, processed, and stimulated with 10 µg/mL recombinant OmpA2 or vehicle control. IFNγ **(a)** and IL-17A **(b)** ELISpots. SFC = spot forming cells. Supernatants collected from stimulated cells were collected and probed for IFNγ **(c)** and IL-17A **(d)** using a LEGENDplex Mouse Th Cytokine Panel (12-plex). Groups were compared using one-way ANOVAs with Tukey post hoc. (*) $p < 0.05$, (**) $p < 0.01$, (***) $p < 0.001$.

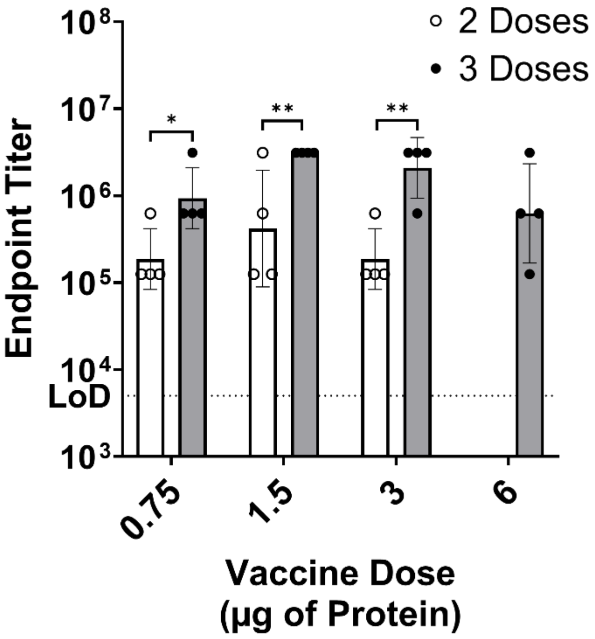


**Fig. S10. AuNP-OmpA2 Dosing Study Antigen-Specific IgG.** C57BL/6 mice were immunized with either 0.75, 1.5, or 3 µg/dose of OmpA2 conjugated to AuNPs and adjuvanted with VacciGrade CpG ODN 2395. Blood was collected one week after the second immunization (d21) and 10 days after the third immunization (d38) was probed for total IgG. Total IgG ELISAs were performed using serial diluted serum and using recombinant OmpA2 as the coating antigen. For comparison, endpoint titers from the high dose vaccination study (6 µg/dose; d42) were also included in the graph. Log transformed endpoint titers were compared using a matched pairs two-way ANOVA with Šidák correction. (*) $p < 0.05$, (**) $p < 0.01$.

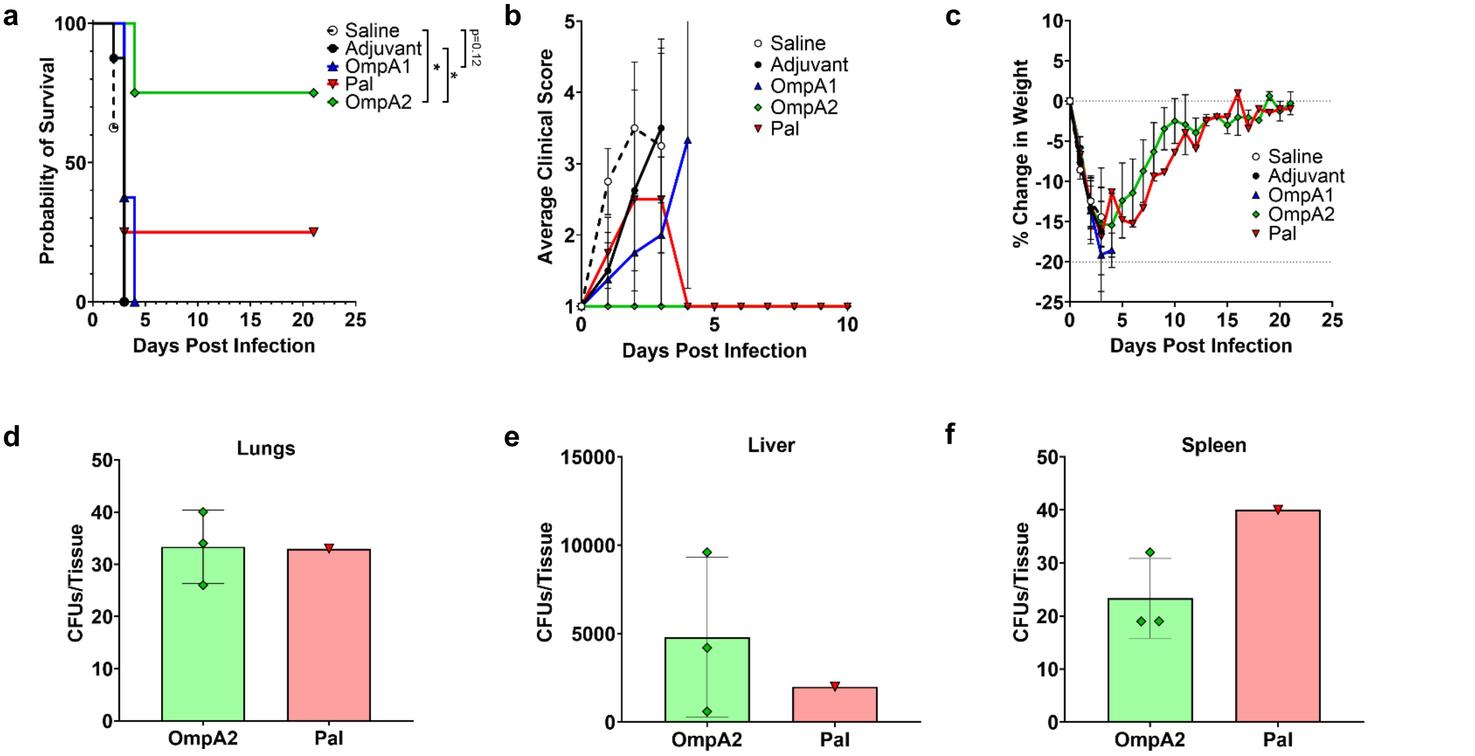


**Fig. S11. High Dose Vaccination and Challenge Study.** C57BL/6 mice were immunized with a high dose of the indicated vaccine containing approximately 6 µg/dose of protein. Three weeks after the third immunization, animals were challenged intranasally with 5.0 x $LD_{50}$ of *Bpm* K96243. **(a)** Survival is reported as a Kaplan-Meier curve. **(b)** Average group clinical score recorded daily up to 10 dpi. Clinical scores were reported as follows: 1 = active and healthy appearance; 2 = mild lethargy; 3 = ruffled fur, hunched posture, and mild lethargy; 4 = ruffled fur, hunched posture, limited mobility; 5 = moribund. **(c)** Percent change in weight from the day of infection. Lungs **(d)**, livers **(e)**, and spleens **(f)** from animals that survived to 21 dpi were homogenized, serially diluted, and plated to assess organ colonization. Survival curves were compared to the saline and adjuvant control groups using log-rank tests and adjusted p values were calculated using the Bonferroni method. (*) $p < 0.05$.

**Table S1: List of Primers.**

| Primer | Sequence |
|---|---|
| BPSL0999 Forward | tgcaccatcatcatcatcatGCAACCCAGCAAGGCACC |
| BPSL0999 Reverse | tggtggtggtggtgctcgagTTACTGCGCCGCTTGCGG |
| BPSL2522 Forward | tgcaccatcatcatcatcatGTTGCTCCGGCCATCACG |
| BPSL2522 Reverse | tggtggtggtggtgctcgagTTACTGCGCCGGAACGGT |
| BPSL2765 Forward | tgcaccatcatcatcatcatAAGTCGGGCGTGAAGCTC |
| BPSL2765 Reverse | tggtggtggtggtgctcgagTTACTGTTGATAGACGAGGTCCG |

**Table S2: List of Targeted Peptides Included in the PRM Assay with Corresponding Precursor m/z Values and Charge States.**

**BPSL0999**

| Peptide | m/z | z |
|---|---|---|
| GAAIGAGVGALVGGVTGYNWQAIK | 744.0708 | 3 |
| NKLAPSAQQTGTQVTEQPDGSLK | 800.0785 | 3 |
| NKLAPSAQQTGTQVTEQPDGSLK | 600.3107 | 4 |
| LAPSAQQTGTQVTEQPDGSLK | 1078.5451 | 2 |
| LAPSAQQTGTQVTEQPDGSLK | 719.3658 | 3 |
| LAPSAQQTGTQVTEQPDGSLK | 539.7762 | 4 |
| AQSVVNALAQR | 578.8253 | 2 |
| GVAANRLSAQGMGASNPIADNATEAGR | 867.0929 | 3 |
| LSAQGMGASNPIADNATEAGR | 1015.9816 | 2 |
| LSAQGMGASNPIADNATEAGR | 677.6568 | 3 |
| LSAQGMGASNPIADNATEAGRAQNR | 834.0701 | 3 |
| LSAQGMGASNPIADNATEAGRAQNR | 625.8044 | 4 |
| RVEIYLR | 474.7849 | 2 |
| VEIYLR | 396.7343 | 2 |
| VEIYLRAPQAAQ | 679.875 | 2 |
| VEIYLRAPQAAQ | 453.5857 | 3 |
| AQSVVNALAQR | 583.8294 | 2 |

**BPSL2522**

| Peptide | m/z | z |
|---|---|---|
| IDEIAAK | 421.5619 | 2 |
| ITYQADTLFDFDK | 788.88166 | 2 |
| QLIACLAPDR | 578.81081 | 2 |
| EKPVALGHDEASWAQNR | 954.4716 | 2 |
| SYLVSKGVPANK | 631.85863 | 2 |
| VEVEVVGTQEVQK | 726.4006 | 2 |
| VEVEVVGTQEVQK | 484.6012 | 3 |
| RVEVEVVGTQEVQK | 533.96062 | 3 |
| RVEVEVVGTQEVQKTTVPAQ | 733.06666 | 3 |
| VEVEVVGTQEVQK | 722.38916 | 2 |

**BPSL2765**

| Peptide | m/z | z |
|---|---|---|
| MHHHHHHACK | 666.2909 | 2 |
| LDEHANQGDAVSTQPNPENVAQVTVDPLNDPNSPLAK | 1947.9407 | 2 |
| LDEHANQGDAVSTQPNPENVAQVTVDPLNDPNSPLAK | 1298.9629 | 3 |
| LDEHANQGDAVSTQPNPENVAQVTVDPLNDPNSPLAK | 974.474 | 4 |
| SVYFDFDSYSVQDQYQALLQQHAQYLK | 1642.7804 | 2 |
| SVYFDFDSYSVQDQYQALLQQHAQYLK | 1098.5227 | 3 |
| SVYFDFDSYSVQDQYQALLQQHAQYLK | 821.8938 | 4 |
| HILIQGNTDER | 648.3388 | 2 |

| | | |
|---|---|---|
| HILIQGNTDER | 432.5616 | 3 |
| GTSEYNLALGQK | 640.8277 | 2 |
| ALSLLGVGDAQMEAVSLGK | 930.0008 | 2 |
| ALSLLGVGDAQMEAVSLGK | 620.3363 | 3 |
| EKPVALGHDEASWAQNR | 954.4716 | 2 |
| EKPVALGHDEASWAQNR | 636.6501 | 3 |
| EKPVALGHDEASWAQNR | 477.7394 | 4 |
| ADLVYQQ | 418.7111 | 2 |
| GTSEYNLALGQK | 644.8384 | 2 |
